# Integrated transcriptome and metabolome provide insight into phenolics and soluble sugar variation in the different varieties of *Gastrodia elata* Blume from different areas in China

**DOI:** 10.3389/fpls.2025.1656554

**Published:** 2025-09-30

**Authors:** Haixia Wang, Shuo Yu, Hanwen Yu, Qingying Fang, Juan Liang, Nannan Zhi, Chengjun Peng, Tingyu Shan, Shuangying Gui, Liangping Zha

**Affiliations:** ^1^ College of Pharmacy, Anhui University of Chinese Medicine, Hefei, China; ^2^ College of Traditional Chinese Medicine, Bozhou University, Bozhou, Anhui, China; ^3^ Center for Xin’an Medicine and Modernization of Traditional Chinese Medicine, Anhui University of Chinese Medicine, Hefei, China; ^4^ MOE-Anhui Joint Collaborative Innovation Center for Quality Improvement of Anhui Genuine Chinese Medicinal Materials, Anhui University of Chinese Medicine, Hefei, China; ^5^ Institute of Pharmaceutics, Anhui Academy of Chinese Medicine, Hefei, China; ^6^ Anhui Province Key Laboratory of Pharmaceutical Preparation Technology and Application, Hefei, China; ^7^ Anhui Engineering Research Center for Quality Improvement and Utilization of Genuine Chinese Medicinal Materials, Anhui University of Chinese Medicine, Hefei, China; ^8^ Institute of Conservation and Development of Traditional Chinese Medicine Resources, Anhui Academy of Chinese Medicine, Anhui University of Chinese Medicine, Hefei, China

**Keywords:** *Gastrodia elata* Blume, gastrodin, metabolome, transcriptome, molecular mechanism

## Abstract

*Gastrodia elata* Blume is known for its "medicinal food homology", its chemical components include phenols, glycosides and polysaccharides. In China, the Anhui, Hubei, Guizhou, and Yunnan provinces are the primary *G. elata*-producing areas, among which *Gastrodia elata* Bl. f. *elata* (GR), *Gastrodia elata* Bl. f. glauca S. Chow (GB), and *Gastrodia elata* Bl. f. *glauca* S. Chow and *Gastrodia elata* Bl. f. *elata* (GR×GB) are essential varieties. This research employed metabolomics and Ultra Performance Liquid Chromatography (UPLC) to quantify 19 soluble sugars and six phenolic compounds in *G. elata* from various origins and varieties. Transcriptome sequencing was performed on 24 *G. elata* samples to identify differentially expressed genes (DEGs). Key enzyme genes involved in the phenolic biosynthesis pathways were further analyzed through phylogenetic analysis, structural modeling and molecular docking. Sucrose, glucose, D-fructose, and D-xylose were identified as the major soluble sugar components in *G. elata*. The phenolic content of the same variety exhibited significant regional differences. In the GR, the largest number of differentially expressed genes (DEGs) was identified in the AH-GR vs. GZ-GR comparison (4,866). In addition, 96 genes encoded 11 key enzymes in the phenolic biosynthesis pathways. In the phenolic synthesis pathway, we identified several alcohol dehydrogenase (ADH) and glucosyltransferase (GT) genes at the downstream level that potentially contribute to the variation in phenolic metabolism. Phylogenetic, structural modeling, and molecular docking analyses suggested that six GTs catalyze the production of gastrodin from p-hydroxybenzyl alcohol. This study provides a fundamental theoretical basis and data support for the selective breeding of *G. elata* varieties and aids in elucidating the regulatory mechanisms of phenolic active compounds.

## Introduction

*Gastrodia elata* Blume is a highly specialized perennial mycoheterotrophic herbaceous plant belonging to the Orchidaceae family. *G. elata* is characterized by the absence of chlorophyll due to its lack of green leaves and primarily derives its nutrients through a symbiotic relationship with *Armillaria mellea* (Vahl) P. Kumm (Yu et al., 2022). *G. elata* is rich in nutrients and various medicinal ingredients, with more than 210 metabolites having been reported in recent years, including phenolic compounds, polysaccharides, glycosides, organic acids, and sterols (Su et al., 2023). Clinically, the dried tubers of *G. elata* are extensively used for the treatment of convulsions, epilepsy, mental disorders, cerebrovascular diseases, inflammation, amnesia, and metabolic disorders (Huang et al., 2024; Zhang et al., 2023; Zeng et al., 2021). Phenolic substances, which are abundant and diverse in *G. elata*, exhibit significant antioxidant activity and are often considered primary contributors to the plant’s pharmacological and therapeutic properties (Heese, 2020). Additionally, polysaccharides derived from *G. elata* have been shown to exert immunomodulatory, antitumor, hypotensive, antibacterial, and free radical-scavenging effects (Zhou et al., 2025; Guan et al., 2022).

*G. elata* has been approved in China as a functional food (Ma et al., 2021). Artificial cultivation technologies have been developed in China, Japan, South Korea, and India (Guo et al., 2016). According to the Flora of China, there are five primary varieties of *G. elata*, with *Gastrodia elata* Bl. f. *elata* (GR), *Gastrodia elata* Bl. f. *glauca* S. Chow (GB) and *Gastrodia elata* Bl. f. *glauca* S. Chow and *Gastrodia elata* Bl. f. *elata* (GR×GB) being the most commonly cultivated (Wu et al., 2009). Moreover, reliable and stable differentiation markers among the GR, GB, and GR×GB have been successfully identified using liquid chromatography-mass spectrometry (Li et al., 2025). The concentration of primary active compounds in a single variety of *G. elata* is influenced by the cultivation region, which includes the geographical environment, soil conditions, and variations in fungal symbiosis and processing methods (Liu et al., 2024; Kang et al., 2017).

Previous studies have applied transcriptome analysis and metabolomics to investigate several metabolic pathways of secondary metabolites in medicinal plants (Saito, 2018), such as *Platycodon grandiflorus* (Jacq.) A. DC (Yu et al., 2021), *Lycium barbarum* L (Ma et al., 2023), and *Taraxacum officinale* (Wieghaus et al., 2022), and compare, using transcriptomics, the differences between the vegetative reproductive corms and white corms of *G. elata* (Tsai et al., 2016). The integration of genome and transcriptome sequencing has established a foundation for investigating secondary metabolic pathways, such as gastrodin (Yuan et al., 2018). Current research on the transcriptome of *G. elata* has primarily focused on the influence of fungal symbiosis on its metabolites (Yu et al., 2022; Zheng et al., 2023). The biological components and nutrients of *G. elata* are influenced by environmental factors and cultivar. Research conducted on the metabolic characteristics of three *G. elata* varieties from the same region revealed that parishins are more prevalent in *G. elata* f. *glauca*, whereas gastrodin, gastrol, and 3,4-dihydroxybenzaldehyde are more abundant in *G. elata* f. *elata* and *G. elata* f. *viridis* (Zeng et al., 2023). The polysaccharide properties of four different *G. elata* species have been extensively studied (Ji et al., 2022). However, data on the differences in gene expression levels in the biosynthetic pathways of phenolics among *G. elata* of different producing areas and varieties remain lacking. Therefore, this study sought to integrate metabolomics and transcriptomics to identify phenolic compounds, soluble sugars, and regulatory genes in three commercially available *G. elata* varieties from four major regions of China, namely Anhui, Hubei, Guizhou, and Yunnan provinces. The findings of this study will enhance our understanding of the biosynthesis of phenolic compounds and soluble sugars in these *G. elata* varieties at the metabolite and gene levels and provide novel insights for the cultivation of *G. elata*.

## Materials and methods

### Plant materials

Two-year-old *Gastrodia elata* Bl. f. *elata* (red stem, GR), *Gastrodia elata* Bl. f. *glauca* S. Chow (black stem, GB), and *Gastrodia elata* Bl. f. *glauca* S. Chow and *Gastrodia elata* Bl. f. *elata* (hybridization of GR and GB, GR×GB) were collected across four Chinese provinces (Anhui, Hubei, Guizhou, and Yunnan), a total of 8 groups, each included three replicates, and each replicate contained one plant. Detailed sampling information is presented in **Supplementary Table S1**. All samples were collected by the same individual in November and were identified by Prof. Huasheng Peng (Anhui University of Chinese Medicine). After collection, they were cleaned of surface soil, blotted dry with filter paper, sliced, immediately flash-frozen in liquid nitrogen, and subsequently stored at –80 °C for further analysis.

### Determination of soluble sugar content

The soluble sugar content and composition were determined using MetWare (Wuhan, China, http://www.metware.cn/). A total of 20 mg powder was diluted to 500 μL methanol: isopropanol: water (3:3:2, v/v/v), vortexed for 3 min, and sonicated for 30 min. The extract was centrifuged at 12,000 rpm under 4 °C for 3 min. The supernatant (50 μL) was mixed with 20 μL internal standard (1000 μg/mL) and evaporated under a nitrogen gas stream. The evaporated samples were transferred to a lyophilizer for freeze-drying. The residue was used for further derivatization. The derivatization method was as follows: the sample was mixed with 100 μL solution of methoxyamine hydrochloride in pyridine (15 mg/mL), incubated at 37 °C for 2 h, and 100 μL of N,O-bis (trimethylsilyl) trifluoroacetamide (BSTFA) was added. The mixture was incubated at 37 °C for 30 min after vortex-mixing. The mixture was analyzed using gas chromatography-mass spectrometry (GC-MS) following dilution to an appropriate concentration (Gómez-González et al., 2010; Medeiros and Simoneit, 2007; Chen et al., 2016).

The Agilent 8890 gas chromatograph coupled to a 5977B mass spectrometer with a DB-5MS column (30 m length × 0.25 mm i.d. × 0.25 μm film thickness; J&W Scientific, USA) was employed for GC-MS analysis of soluble sugars. Helium was used as a carrier gas at a flow rate of 1 mL/min. Injections were made, with an injection volume of 1 μL, in split mode with a split ratio of 5:1. The oven temperature was held at 160 °C for 1 min, and then raised to 200 °C at 6 °C/min, 270 °C at 10 °C/min, 300 °C at 5 °C/min, and finally 320 °C at 20 °C/min and held for 5.5 min. All samples were analyzed in the selective ion monitoring mode. The ion source and transfer line temperatures were 230 °C and 280 °C, respectively (Medeiros and Simoneit, 2007; Chen et al., 2016). Principal component analysis (PCA) and hierarchical cluster analysis (HCA) were used to analyze the accumulation patterns of the metabolomic data. The HCA results of the samples and metabolites are presented as heat maps accompanied by dendrograms. The fold change (FC) ≥ 2 or fold change (FC) ≤ 0.5 was set as the screening condition for differentially accumulated metabolites (DAMs).

### Determination of phenolic content

As previously described, with minor modification, the phenolic content was extracted from dried *G. elata* samples and measured (Chen et al., 2016). Briefly, the dried powder (0.5 g) of each *G. elata* sample was extracted in 25 mL of 50% methanol via ultrasonication for 2 h. The phenols in the extract were chromatographed and detected using an Acquity-UPLC™ (Waters, MA, USA) system equipped with a quaternary pump, degasser, autosampler, and photodiode array detector (PDA). Phenols were separated on an ACQUITY UPLC^®^ T3 column (1.8 μm, 2.1 mm × 100 mm) (Waters, MA, USA). The mobile phase consisted of water containing 0.05% phosphoric acid (A) and acetonitrile (B) with a flow rate of 0.2 mL/min at 25 °C. The linear gradient elution program was as follows: 0~2 min, 5%~7% B; 2~3 min, 7%~10% B; 5~7 min, 20%~24% B; 7~9 min, 15%~18% B; 9~12 min, 18%~20% B; 12~14 min, 80%~76% B; 14~19 min, 24%~27% B; 19~21 min, 27%~15% B; 21~24 min, 15%~5% B. The injection volume was 2 μL, and the chromatograms were acquired at 220 nm.

### RNA extraction and illumina sequencing

AH-GR, HB-GR, GZ-GR, AH-GR×GB, HB-GR×GB, YN-GR×GB, GZ-GB and YN-GB were used for RNA-seq analysis. Total RNA was extracted by ethanol precipitation and CTAB-PBIOZOL, and genomic DNA was removed from the total RNA using DNase I. Subsequently, total RNA was identified and quantified using a Qubit fluorescence quantifier and Qsep400 high-throughput biofragment analyzer. The cDNA libraries were constructed using MetWare Biotechnology Co., Ltd. (Wuhan, China). The final library was amplified with phi29 before being uploaded to make DNA nanoballs (DNB) with more than 300 copies of a molecule, and the DNBs were loaded onto the sequencing chip and sequenced on the BGI sequencing platform.

### Functional annotation

To obtain high-quality clean data, fastp software was used for strict quality control of the data (Chen et al., 2018). The adapters of reads are removed simultaneously, along with low-quality reads (including those with an N ratio greater than 10% and those with Q ≤ 20 bases accounting for more than 50% of the entire read). The HISAT2 software was used to compare the clean reads with the reference genome to obtain localization data of the reads on the reference genome (Kim et al., 2015). StringTie was used to assemble the reads and reconstruct the transcriptomes for subsequent analyses (Pertea et al., 2015). Finally, annotation data were obtained by sequence alignment of the genes with the Kyoto Encyclopedia of Genes and Genomes (KEGG), Gene Ontology (GO), Non-Redundant Protein Database (NR), Swiss Protein Database (Swiss-Prot), Translated European Molecular Biology Laboratory Nucleotide Sequence Database (TrEMBL), and EuKaryotic Orthologous Groups (KOG) databases using Diamond, with an E-value of 1e^-5^.

### Differential expression analysis

The Stringtie maximum flow algorithm and fragments per kilobase of transcript per million fragments mapped (FPKM) were used to calculate gene expression levels. DESeq2 (Love et al., 2014) was used for differentially expressed genes (DEGs) screening based on the count values of genes in each sample. The false discovery rate (FDR) was obtained by correcting the probability of hypothesis testing (P-value) for multiple hypothesis testing using the Benjamini-Hochberg method. DEGs were screened for Fold Change ≥ 2 and FDR < 0.01. The total numbers of DEGs, upregulated genes, and downregulated genes in each group were counted and visualized using OmicShare tools (https://www.omicshare.com/tools/) and EVenn (http://www.ehbio.com/test/venn/#/).

### Phylogenetic analysis

Phylogenetic trees were constructed with MEGA 6.0 software using the neighbor-joining method with 1,000 bootstrap replicates. The amino acid sequence of the UGT from *Arabidopsis thaliana* was obtained from the CAZy database (http://www.cazy.org/) (Ross et al., 2001). Several UGT proteins that have been confirmed to encode the function of catalyzing the conversion of p-hydroxybenzyl alcohol to gastrodin in *Escherichia coli* and *Saccharomyces cerevisiae* were used to construct phylogenetic trees, namely UGT73B6 (GenBank: AY547304) (Bai et al., 2014, 2016; Zhao et al., 2021), UGT73B6FS (Bai et al., 2016), AsUGT (GenBank: Q9AR73) (Yin et al., 2022), AsUGTsyn (Yin et al., 2022), AtUGT (GenBank: KAG7614719.1) (Xia et al., 2024), CtUGT (GenBank: AJT58578.1) (Xia et al., 2024), PgUGT (GenBank: AIE12483.1) (Xia et al., 2024), and RgUGT (GenBank: KAG5565971.1) (Xia et al., 2024). The constructed phylogenetic tree was visualized and identified using the iTOL tool (https://itol.embl.de/).

### Sequence alignment, structure modeling, and molecular docking analyses

Multiple sequence alignments were performed using the ESPript online program (https://espript.ibcp.fr/ESPript/cgi-bin/ESPript.cgi). A three-dimensional model of GeUGTs was generated using the SWISS-MODEL server and visualized using PyMOL 3.0. Molecular docking was performed using AutoDock 1.5.7 to predict the binding of the glycosyl donor UDPG to the GeUGT protein, thereby forming the GeUGT-UDPG complex. Subsequently, p-hydroxybenzyl alcohol was docked onto the GeUGT-UDPG complex. The protein structure modeling and molecular docking are outlined in **Supplementary Tables S2** and **S3**.

### Quantitative real-time polymerase chain reaction analysis

To validate the reliability of the RNA-seq results for determining gene expression, qRT-PCR was performed. Based on our preliminary studies and literature survey, the β-actin gene was selected as the reference gene (Yuan et al., 2018; Shan et al., 2021). Twelve DEGs involved in phenolic compound and carbohydrate metabolism were selected for qRT-PCR validation. Gene-specific primers were designed using the Primer 5.0 software and synthesized by General Biology Co., Ltd. (Anhui, China; **Supplementary Table S4**). qRT-PCR analysis was conducted using the BioRad CFX96 Real-Time PCR Detection System (Bio-Rad Laboratories) following the manufacturer’s protocol (LABLEAD, China), including optimized cycling conditions and reaction mixture proportions. Relative gene expression levels were quantified using the 2^−ΔΔCt^ method. All experiments were performed in triplicate to ensure data reproducibility.

### Data statistical analysis

Microsoft Excel and GraphPad Prism 9.30 software were used to statistically analyze the data. The statistical analysis method used was one-way ANOVA. The TB tools software 2.331, Metware Cloud (https://cloud.metware.cn), and OmicShare tools (https://www.omicshare.com/tools/) were used for visualization and analysis.

## Results

### Morphological variations and soluble sugar analysis in *G. elata*

**Figure 1:**
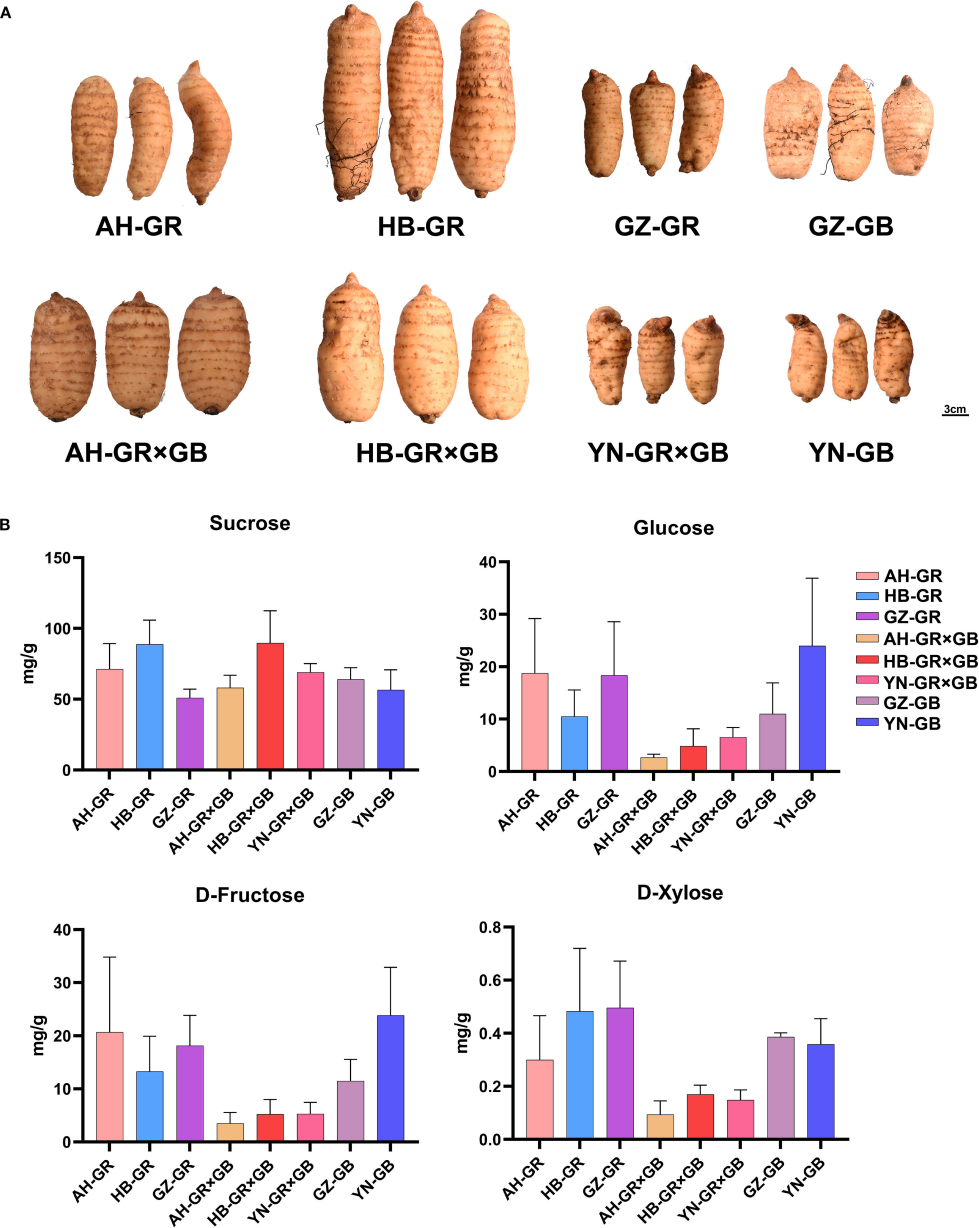
Morphological characteristics of three *G. elata* cultivars and the statistical analysis of soluble sugar contents from *G. elata*. **(A)** The morphological characteristics of *G. elata*. Scale bars = 3 cm. **(B)** Sucrose, Glucose, D-fructose, and D-xylose contents from different producing areas and varieties of *G. elata*.

The morphology and appearance of *G. elata* tubers vary considerably across different cultivars. The GR exhibits an elongated cylindrical shape, whereas the GB is characterized by a distinct oval form. The GR×GB effectively combines the favorable morphological traits of both parental lines, demonstrating a more robust and plump tuber morphology than its parents (**Figure 1A**). Soluble sugars in *G. elata* tubers from different producing areas and cultivars were analyzed using GC-MS. The total ion overlap diagram chromatograms demonstrated excellent instrument stability (**Supplementary Figure S1A**). PCA revealed a clear separation among the samples (**Supplementary Figure S1B**). A total of 19 soluble sugar components were identified (**Supplementary Table S5**), among which sucrose, glucose, D-fructose, and D-xylose were the major soluble sugar components in *G. elata*, collectively accounting for over 98% of the total soluble sugar content. Sucrose (50.91–80.81 mg/g) was the most abundant across all samples (**Supplementary Table S5**). Comparatively, the content of glucose, D-fructose, and D-xylose in GR and GB was higher than that in GR×GB (**Figure 1B**). DAMs primarily consisted of monosaccharides and disaccharides. Specifically, 3, 5, 3, 1, 3, and 3 DAMs were detected in the comparison groups HB-GR vs. AH-GR, GZ-GR vs. AH-GR, GZ-GR vs. HB-GR, HB-GR×GB vs. AH-GR×GB, AH-GR×GB vs. YN-GR×GB, and YN-GB vs. GZ-GB, respectively (**Supplementary Table S6**), and identified a DAM (xylitol) in the GR from three regions (**Supplementary Figure S2**). Additionally, in the comparative groups from the same regions of Anhui, Hubei, Guizhou, and Yunnan, there were 7, 3, 3, and 8 DAMs (**Supplementary Table S6**), respectively.

### Analysis of phenolic metabolites in *G. elata*

**Figure 2:**
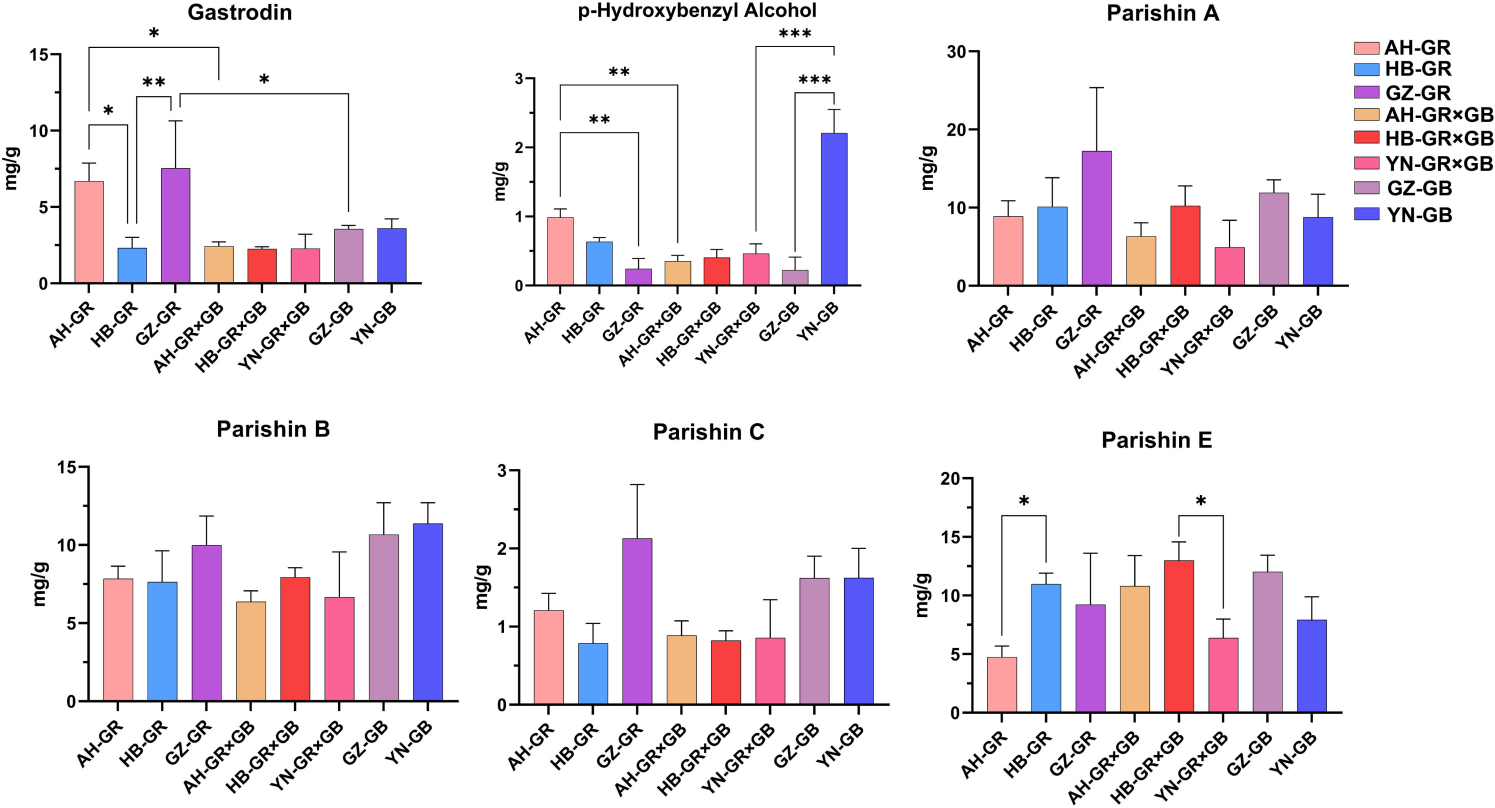
Contents of phenolic in *G. elata* cultivars. ’*’ represents statistical differences in the same content between different cultivars, with *p <* 0.05 being a significant difference (*p < 0.05, **p < 0.01, ***p < 0.001). Error bars represent standard deviations.

Phenolic compounds are the major class of bioactive constituents in *G. elata* (Shan et al., 2021). In all examined samples, parishin A, parishin B, and parishin E were present at relatively high concentrations, whereas p-hydroxybenzyl alcohol and parishin C were detected at comparatively lower levels (**Supplementary Table S7**). In the GR group, the gastrodin content in AH-GR and GZ-GR was significantly higher (*p* < 0.05) than that in HB-GR. The p-hydroxybenzyl alcohol content in AH-GR was significantly higher than that in GZ-GR, while the parishin E content in HB-GR was significantly higher (*p* < 0.05) than that in AH-GR. In the GR×GB group (**Figure 2**), significant differences were observed in the contents of parishin A and parishin E between the HB-GR×GB and YN-GR×GB. In the GB group, the content of p-hydroxybenzyl alcohol in YN-GB accessions was higher than that in GZ-GB (**Figure 2**). Differences were also observed in the content of specific compounds among different varieties of *G. elata* from the same origin. The gastrodin and p-hydroxybenzyl alcohol content in AH-GR were significantly higher (*p* < 0.05) than those in AH-GR×GB, the gastrodin content in GZ-GR was significantly higher than that in GZ-GB, and the p-hydroxybenzyl alcohol content in YN-GB was significantly higher than that in YN-GR×GB (**Figure 2**).

### Transcriptomic differences reflect regional and varietal influences

A total of 24 cDNA libraries were subjected to transcriptome sequencing and yielded between 43,248,992 and 56,780,560 clean reads; the Q30 and Q20 percentages for all libraries exceeded 92.54% and the GC content percentages were > 45.43% (**Supplementary Table S8**). The correlation coefficient between samples exceeded 0.85 (**Supplementary Figure S3**). The PCA plot shows that PC1 and PC2 account for 26.4% and 18.1% of the variance, respectively (**Supplementary Figure S4**). Overall, the results of transcriptome analysis were of high quality and suitable for further research. Of the 23,162 identified genes, 18,415 (79.51%) were successfully annotated using at least one of the following databases: Nr, SwissPort, KOG, Trembl, Pfam, GO, or KEGG (**Supplementary Table S9**; **Supplementary Figure S5**). Among the 18,347 genes annotated in the NR database, 87.88% showed homologous sequence matches with eight species of the Orchidaceae family, with the highest homology observed with *Dendrobium catenatum* (**Supplementary Figure S6**). Among the 15,504 genes annotated with GO terms, the categories were predominantly “cellular process” and “metabolic process” in biological processes, “cellular anatomical entity” in cellular components, and “binding” and “catalytic activity” in the molecular function categories (**Supplementary Figure S7**). Additionally, 14,161 genes were mapped to 147 pathways using KEGG annotation (**Supplementary Figure S8**).

**Figure 3:**
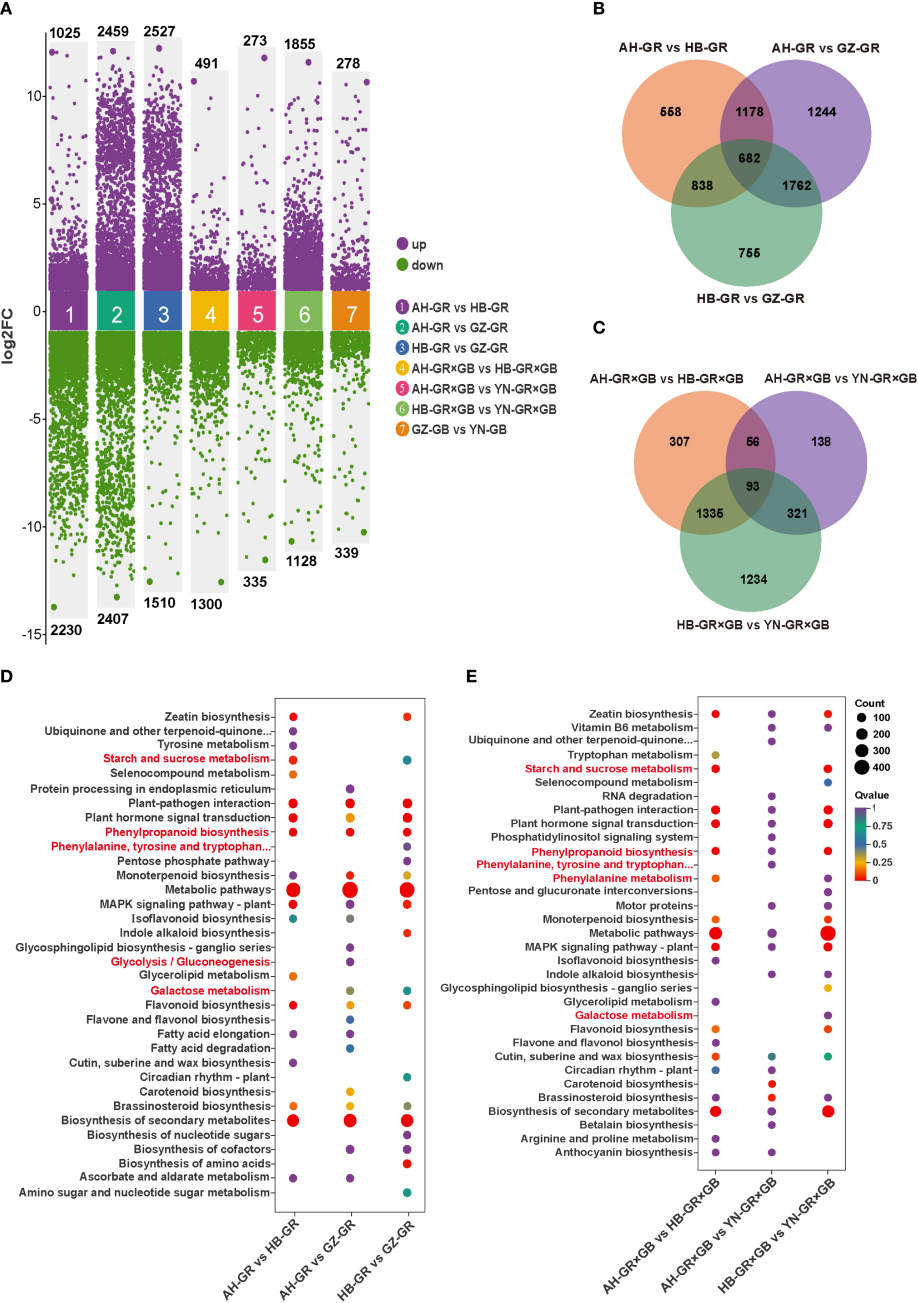
Different expression genes (DEGs) of three commercially available *G. elata* varieties from four major regions. **(A)** DEGs statistics of AH-GR vs. HB-GR, AH-GR vs. GZ-GR, HB-GR vs. GZ-GR, AH-GR×GB vs. HB-GR×GB, AH-GR×GB vs. YN-GR×GB, HB-GR×GB vs. YN-GR×GB, and GZ-GB vs. YN-GB. Purple represents upregulated DEGs, green represents downregulated DEGs. **(B)** The Venn diagram of DEGs in GR. **(C)** The Venn diagram of DEGs in GR×GB. **(D)** The top 20 enriched KEGG pathways of DEGs in AH-GR vs. HB-GR, AH-GR vs. GZ-GR, and HB-GR vs. GZ-GR. **(E)** The top 20 enriched KEGG pathways of DEGs in AH-GR×GB vs. HB-GR×GB, AH-GR×GB vs. YN-GR×GB, and HB-GR×GB vs. YN-GR×GB. The size of the dots represented the number of DEGs. Red and purple represented high and low expression levels, respectively.

The analysis also revealed that the number of DEGs in the three pairwise comparisons within the GR group from different producing areas was substantially higher than that in the GR×GB and GB groups, reaching 3,255, 4,866 and 4,037, respectively. The GR×GB groups contained 1,791, 608 and 2,983 DEGs, while the GB group showed the lowest number, with a total of only 617 (**Figure 3A**). Furthermore, the number of DEGs in the four comparison groups of different varieties within the same origin was generally low. Particularly, the comparison between HB-GR×GB and HB-GR yielded only common 50 DEGs (**Supplementary Figure S9**). Based on the identified differences from the comparisons, we conducted further analysis on *G. elata* of the same variety from different producing areas. Venn diagrams illustrated 682 common DEGs in the three GR comparison groups (**Figure 3B**), and 93 in the three GR×GB comparison groups (**Figure 3C**). Further analysis of the DEGs across different groups was conducted to investigate their functional categories using the KEGG database. The highest number of DEGs was identified in the comparison AH-GR_vs._GZ-GR within the GR group (**Figure 3D**), with an enrichment of 139 KEGG pathways. Conversely, the fewest DEGs were identified in the AH-GR×GB_vs._YN-GR×GB within the GR×GB group (**Figure 3E**), with 159 DEGs enriched in 93 KEGG pathways. In the GB comparison group, GZ-GB_vs._YN-GB (**Supplementary Figure S10**) revealed enrichment of 177 DEGs across 102 KEGG pathways. Seven comparison groups demonstrated significant enrichment in the “metabolic pathway,” “secondary metabolite biosynthesis,” and “plant-pathogen interaction” pathways. Notably, pathways related to phenolic metabolism, such as phenylpropanoid biosynthesis, phenylalanine metabolism, and the biosynthesis of phenylalanine, tyrosine, and tryptophan, as well as glucose metabolic pathways, including starch and sucrose metabolism, galactose metabolism, and glycolysis/gluconeogenesis, were enriched.

### Unraveling the phenolic biosynthesis in *G. elata* through a multi-omics approach

**Figure 4:**
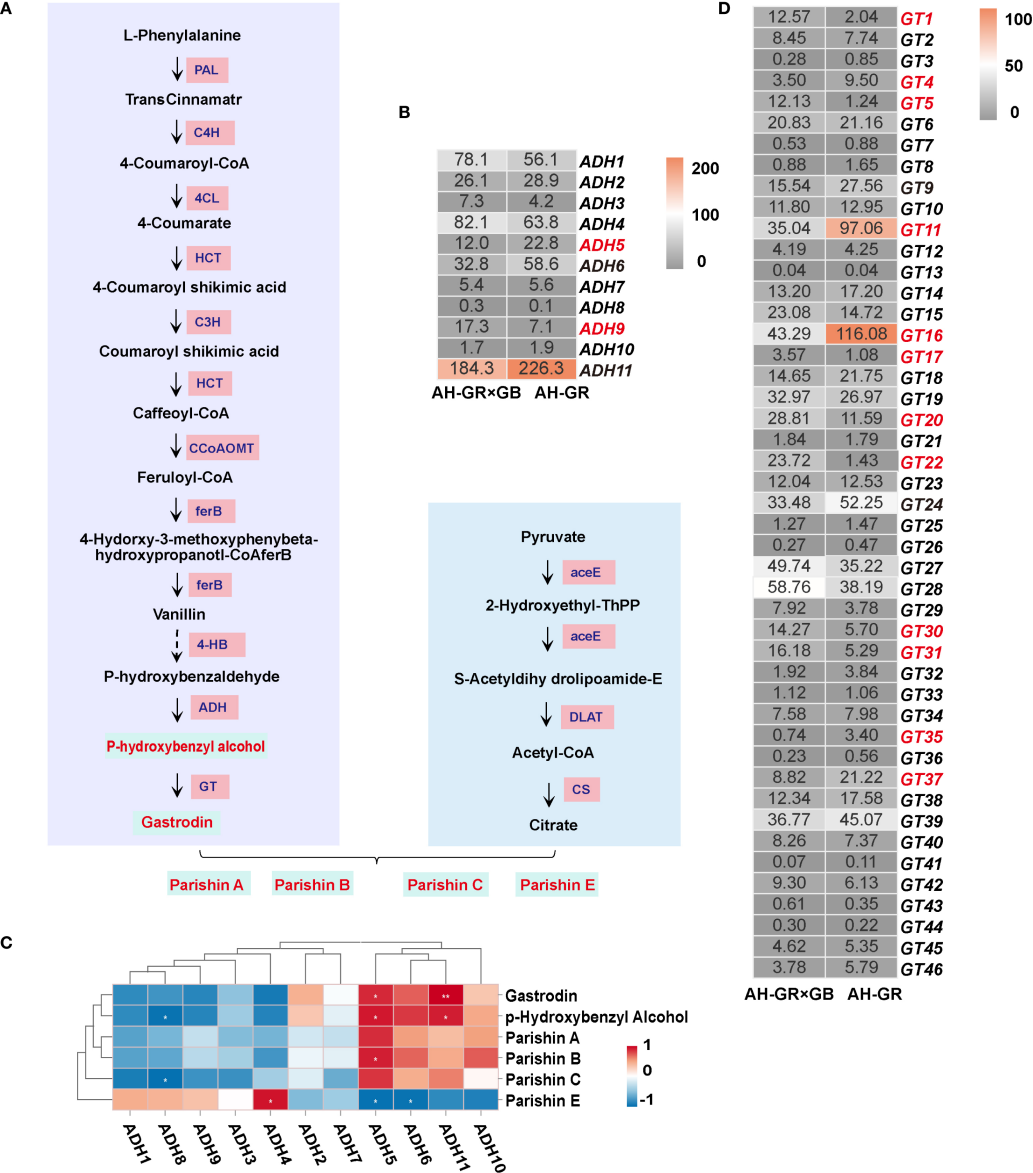
Integrated transcriptomic and metabolomic analysis of the phenolic biosynthesis pathway. **(A)** The downstream GT genes with the FPKM; the red font indicates the DEGs in AH-GR and AH-GR×GB. **(B)** The putative pathway of phenolic biosynthesis in *G. elata.* **(C)** The downstream ADH genes with the FPKM; the red font indicates the DEGs in AH-GR and AH-GR×GB. **(D)** Correlation heatmap between 11 ADH genes and 6 kinds of phenols (*p < 0.05, **p < 0.01, ***p < 0.001).

In our previous study, we postulated the biosynthetic pathway of phenolic compounds in *G. elata* (Shan et al., 2021) (**Figure 4B**). Phenolic metabolite analysis in *G. elata* revealed significant variations in phenolic content across different producing areas and varieties. Based on these findings, we next investigated the molecular mechanisms underlying the differential accumulation of phenolic compounds in *G. elata*. Gastrodin, p-hydroxybenzaldehyde, and p-hydroxybenzyl alcohol are synthesized from phenylalanine via the phenylpropanoid pathway, whereas the citric acid biosynthesis pathway is implicated in the biosynthesis of parishin, we identified a total of 11 enzymes: Phenylalanine ammonialyase (PAL), Cinnamate4-hydroxylase (C4H), 4-comarate coenzymeA ligase (4CL), Shikimate O-hydroxycinnamoyltransferase (HCT), 5-O-(4-coumaroyl)-D-quinate3’-monooxygenase (C3H), Caffeoyl-CoA O-methyltransferase (CCoAOMT), Alcoholdehydrogenase (ADH), Glycosyltransferases (GT), Pyruvatedehydrogenase/pyruvate dehydrogenase complex (aceE), Dihydrolipoyllysine-residue acetyltransferase (DLAT) and Citrate symthase (CS) corresponding to 96 genes (**Supplementary Table S10**). The phenylpropanoid biosynthesis pathway serves as the fundamental route for the synthesis of various phenolic compounds in plants, with its upstream steps primarily responsible for generating universal precursors. For our target metabolites, that is, gastrodin, p-hydroxybenzyl alcohol, and parishin-type compounds, the downstream ADH and GT genes represent more critical targets for investigation. The expression of *ADH1* and *ADH4* was lower in AH-GR than AH-GR×GB, whereas the expression of *ADH5*, *ADH6*, and *ADH11* was higher (**Figure 4B**), which may be attributed to the higher content of p-hydroxybenzyl alcohol and gastrodin in AH-GR. Correlation clustering analysis between six phenolic compounds and eleven ADH genes indicated a significant positive correlation (*p* < 0.05) of *ADH5* and *ADH11* with the accumulation of p-hydroxybenzyl alcohol and gastrodin (**Figure 4C**), suggesting that *ADH5* and *ADH11* are likely key genes involved in the biosynthesis of p-hydroxybenzyl alcohol. The content of gastrodin and p-hydroxybenzyl alcohol in AH-GR was significantly higher than that in AH-GR×GB (**Figure 2**). We identified a total of 46 GT genes; there were 12 differentially expressed GTs (*GT1*, *GT4*, *GT5*, *GT11*, *GT16*, *GT17*, *GT20*, *GT22*, *GT30*, *GT31*, *GT35*, and *GT37*) between AH-GR×GB and AH-GR (**Figure 4D**).

### Correlation and phylogenetic analysis of GeUGTs

**Figure 5:**
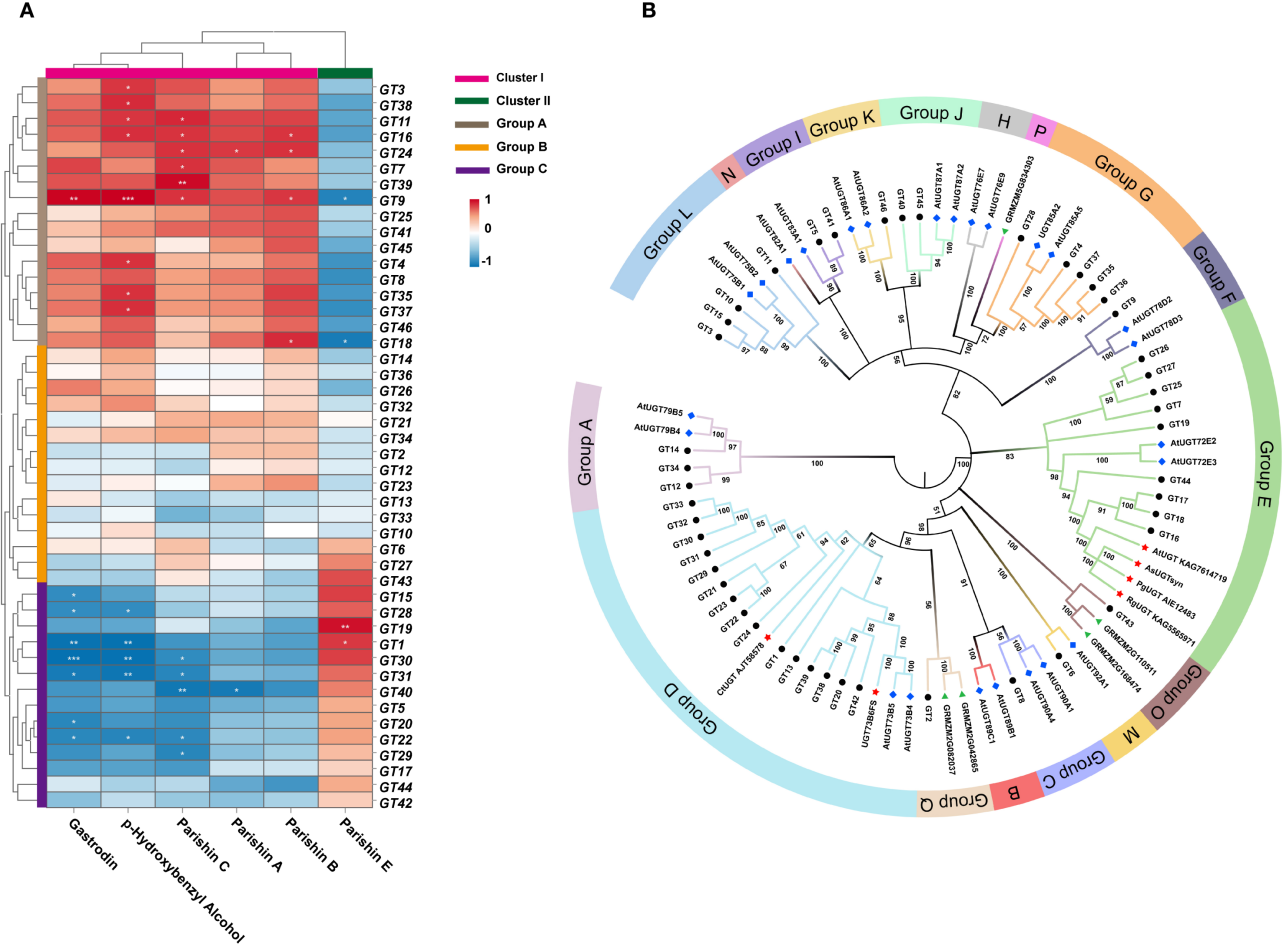
**(A)** Correlation clustering analysis between 6 phenolic compounds and 46 GT genes (*p < 0.05, **p < 0.01, ***p < 0.001). **(B)** Phylogenetic tree of the UGT family members. Black circular dots represent *G. elata* proteins, blue rhombuses represent Arabidopsis proteins, green triangles represent maize proteins, and red five-pointed stars represent proteins with known function.

Correlation clustering analysis between six phenolic compounds and 46 GT genes revealed (**Figure 5A**) that Group A (*GT3*, *GT38*, *GT11, GT16*, *GT24*, GT*7*, *GT39*, *GT9*, *GT25*, *GT41*, *GT45*, *GT4, GT8*, *GT35*, *GT37*, *GT46*, and *GT18*) and Cluster I (parishin A, parishin B, parishin C, p-hydroxybenzyl alcohol, and gastrodin), which showed positive correlations (*p* < 0.05), negatively correlated with Cluster II (parishin E). In Group C, *GT19* and *GT1* significantly positively correlated with parishin E (*p* < 0.05). These findings provide key candidate genes and a solid basis for further functional studies on glycosyltransferases in *G. elata*. To gain a comprehensive understanding of the evolutionary relationships and classify the 46 UGT proteins in *G. elata*, a phylogenetic tree including 25 UGT proteins from *Arabidopsis thaliana* (Groups A to N) and 5 from *Zea mays* (Groups O to Q) was constructed using MEGA 7.0. The phylogenetic tree revealed that the UGT proteins in *G. elata* could be classified into 13 groups, with no sequences identified in groups B, H, N, or P (**Figure 5B**). Among them, group D contained the largest number of UGT proteins of *G. elata*, with a total of 15 members, followed by group E, which contained 9 members. Groups G, L, and A consisted of five, four, and three members, respectively. Groups C, F, K, M, and O each contained only one member. Notably, group D contained two UGT members (CtUGT and UGT73B6FS), whereas group E contained 4 UGT members (AtUGT, AsUGTsyn, PgUGT, and RgUGT), all of which have been shown to catalyze the conversion of p-hydroxybenzyl alcohol to gastrodin *in vitro*. The 24 UGT genes in groups D and E of *G. elata* may also have similar catalytic functions.

### Sequence alignment, structure modeling and molecular docking analysis

**Figure 6:**
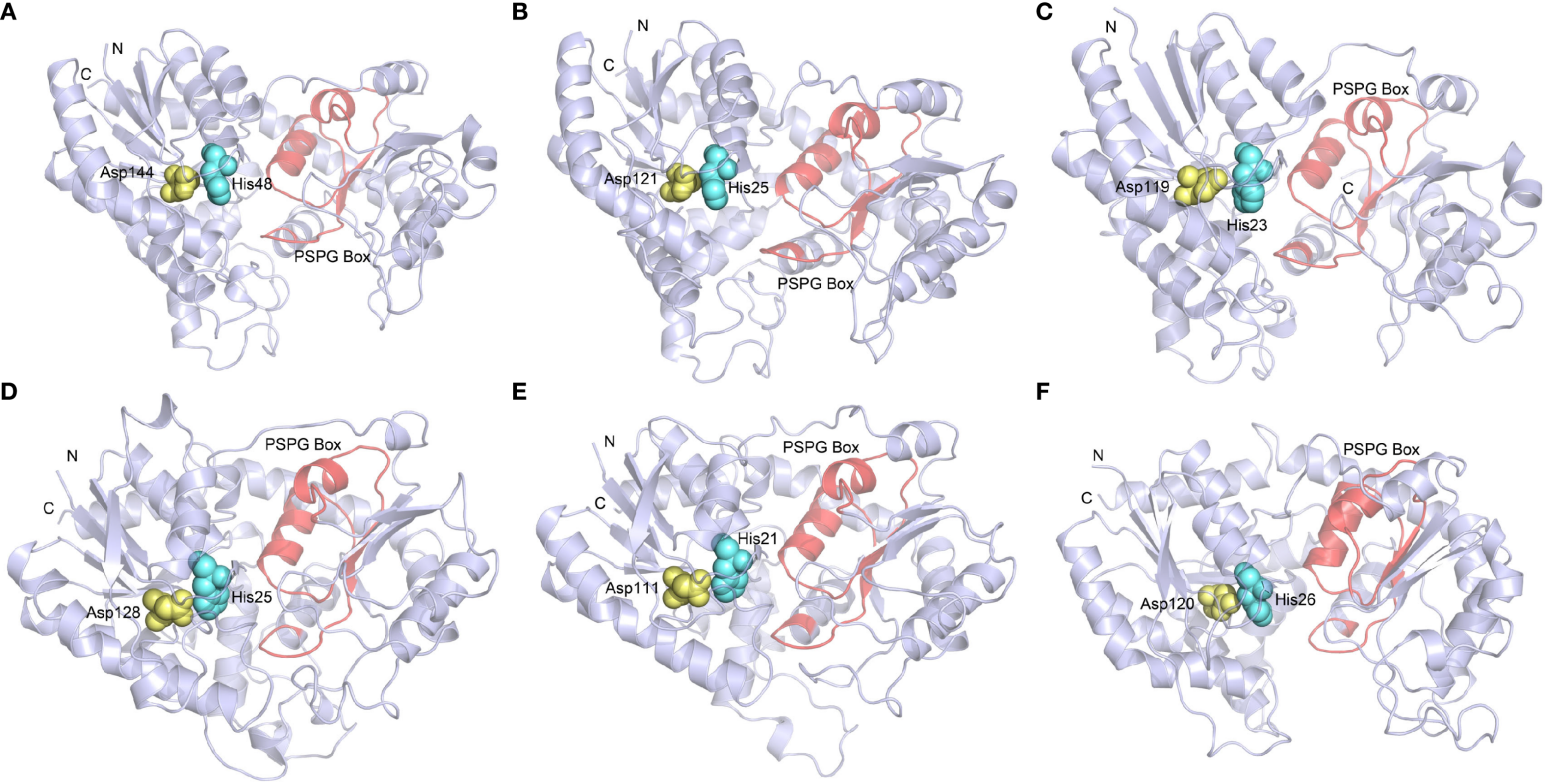
Spatial structure model of GT16 **(A)**, GT17 **(B)**, GT18 **(C)**, GT24 **(D)**, GT42 **(E)**, and GT44 **(F)**.

Based on the phylogenetic analysis, four GeUGTs (GT16, GT17, GT18, and GT44) from group E and two GeUGTs (GT24 and GT42) from group D, which showed close evolutionary relationships with functionally characterized UGT genes, were selected for sequence and structural analyses. Multiple sequence alignments revealed that these six GeUGTs shared 48.81% sequence identity with the known functional UGTs. All identified GeUGTs contained a highly conserved 44-amino acid sequence at their C-termini, designated the plant secondary product glycosyltransferase (PSPG) motif, which is characteristic of plant secondary metabolism glycosyltransferases. Notably, the catalytic residues His (H) and Asp (D) are highly conserved among the UGTs (**Supplementary Figure S11**). Subsequent tertiary structure modeling demonstrated that all six GeUGT structures were monomeric proteins. In the structural models, the PSPG box is highlighted in red, while the catalytic residues are represented as spheres, with histidine (His) in blue and aspartate (Asp) in yellow (**Figure 6**).

**Figure 7:**
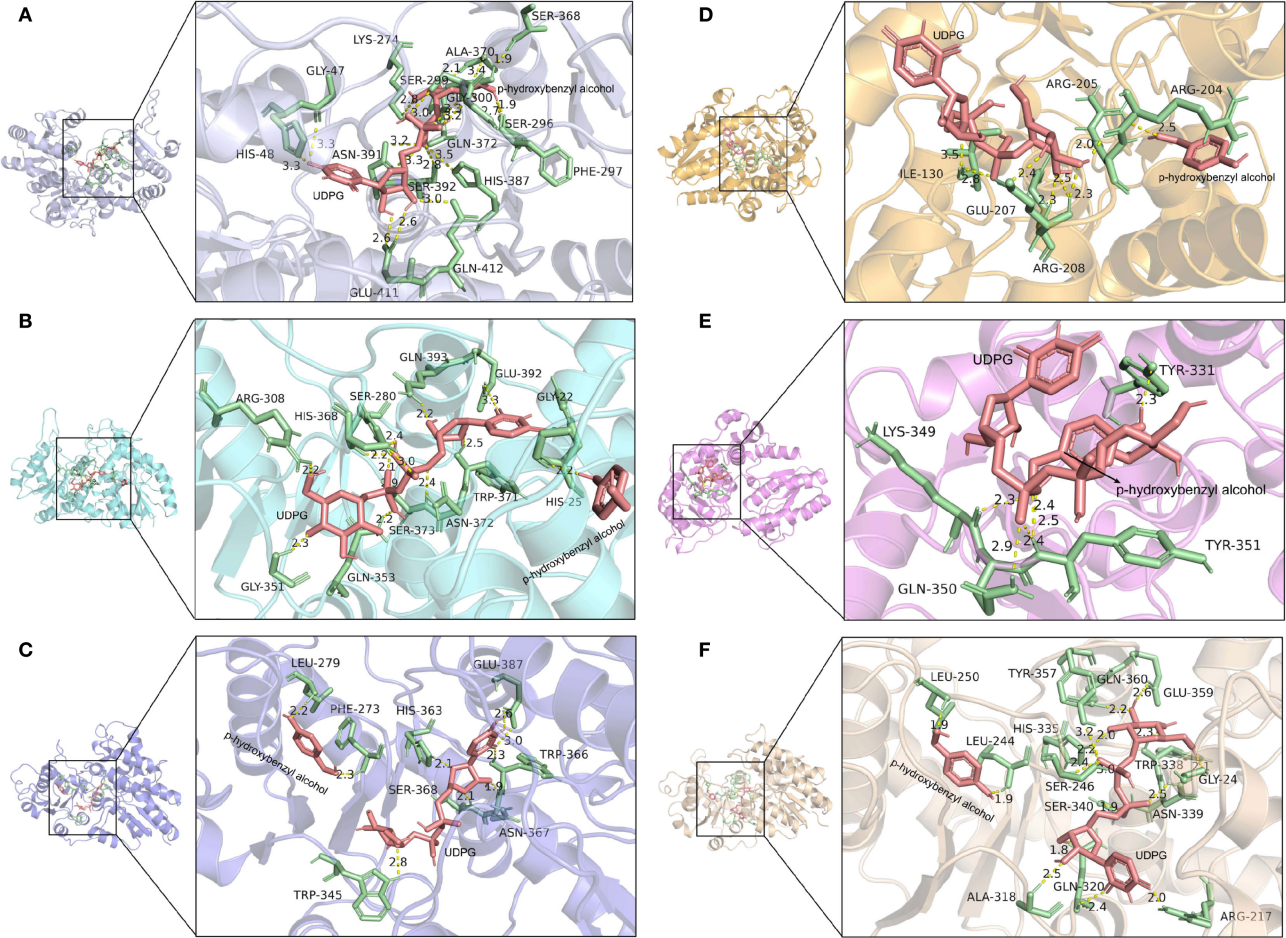
Visualization of the molecular docking results of GT16 **(A)**, GT17 **(B)**, GT18 **(C)**, GT24 **(D)**, GT42 **(E)**, and GT44 **(F)**.

Molecular docking was used to investigate the interaction between the protein and ligand and predicted the stability and binding affinity of the docked complex (**Supplementary Table S11**). The molecular docking analysis revealed that p-hydroxybenzyl alcohol binds to the GT16-UDPG complex with a binding energy of -5.043 kcal/mol. Additionally, His48 formed hydrogen bonds with UDPG at a distance of 3.3 Å (**Figure 7A**). Similarly, p-hydroxybenzyl alcohol exhibited stronger binding affinity (-5.326 kcal/mol) to the GT17-UDPG complex, where the catalytic residue His25 formed shorter hydrogen bonds (2.2 Å) with UDP-glucose (**Figure 7B**). The key conserved residues in the PSPG box of GT16, GT17, GT18, and GT44 formed hydrogen bonds with UDP-glucose (UDPG), facilitating substrate recognition and binding (**Figures 7A-C, F**). However, in GT24 and GT42, neither the catalytic residue His nor the key residues within the PSPG box formed interactions with UDPG and p-hydroxybenzyl alcohol (**Figures 7D, E**).

### qRT-PCR analysis

To confirm the accuracy of the transcriptome sequencing results, six DEGs (*PAL4*, *CCoAMT1*, *C4H*, *4CL2*, *ADH4*, *UGE1*, and *INV8*) involved in phenolic and soluble sugar metabolic pathways, and six GT genes (*GT16*, *GT17*, *GT18*, *GT24*, *GT42*, and *GT44*) were randomly selected for validation using qRT-PCR analysis. As shown in **Supplementary Figure S12**, the expression profiles of the 12 genes were consistent in the qRT-PCR and FPKM values, confirming the accuracy and repeatability of the transcriptome analysis.

## Discussion

The accumulation of secondary metabolites and quality traits in medicinal plants is co-regulated by the genetic characteristics of germplasm resources and geo-ecological factors, such as climate conditions, soil physicochemical properties, and altitude gradients (Li et al., 2020). Regional environments, cultivation techniques, and management practices significantly influence the growth of *G. elata*. With breakthroughs in planting technologies and the rapid development of the health industry, the cultivation area of *G. elata* has expanded, both globally and nationally, garnering significant attention for the breeding of varieties adapted to different regions (Fan et al., 2025). Zhong collected *G. elata* from Yunnan, Guizhou, and Hubei provinces to investigate its volatile components. The authors showed that the flavor characteristics of *G. elata* from Yunnan were significantly superior to those from Guizhou and Hubei (Zhong et al., 2025). In this study, the samples were sourced from partial traditional producing areas spanning two major regions: Southwest China (Yunnan and Guizhou) and Central China (Hubei and Anhui). Soluble sugars are important nutritional components in *G. elata*. Sucrose, glucose, D-fructose, and D-xylose were the most abundant in all *G. elata* samples (**Figure 1B**), however, their variations across different producing areas and varieties were not significant. Therefore, we focused more on the differences in medicinal components, specifically phenolic compounds. Among different varieties of *G. elata*, the phenolic content was highest in the GB, followed by GR, and lowest in the GR×GB. From a geographical perspective, the phenolic content of the same variety shows significant regional differences, consistent with previous findings (Zhu et al., 2024).

Previous studies compared the transcriptomic differences between the tubers and immature tubers of *G. elata*, identifying two unigenes potentially involved in the hydroxylation and glycosylation steps of gastrodin biosynthesis (Tsai et al., 2016). Transcriptomic analysis has also been conducted on the flowers, stems, and tubers of *G. elata*, along with a comparison of the content of six major phenolic compounds (Shan et al., 2021). Another study performed metabolomic and transcriptomic analyses on immature and mature tubers of *G. elata*, detecting 76 different amino acids and their derivatives as well as 34 vitamins in the metabolome. A total of 16 DEGs were identified in the transcriptome, and the enzymes encoded by these DEGs were found to promote or inhibit the biosynthesis of amino acids and vitamins in *G. elata* (Wang and Shahid, 2023). Additionally, research on the transcriptome of *G. elata* seeds during germination in symbiosis with mycorrhizal fungi revealed the involvement of clathrin-mediated endocytosis in seed germination and elucidated the molecular mechanisms of the symbiotic germination between endogenous fungi and *G. elata*. These findings provide significant guidance for the innovative development of *G. elata* cultivation techniques (Zeng et al., 2017). However, transcriptomic data for *G. elata* from different regions and varieties remain lacking. Some studies have attributed the regional differences in metabolic accumulation and antioxidant capacity of mature *G. elata* tubers to the genetic characteristics of germplasm resources and geoecological factors (Zhang et al., 2024). Our transcriptome analysis of eight groups of *G. elata* from different origins and varieties showed a lower number of DEGs within the same production area, and a higher number for the same variety across different production areas (**Supplementary Figure S9**). Furthermore, 93 common DEGs were identified in the GR×GB comparison across three production areas (**Figure 3B**), indicating genetic stability in the selected hybrid variety during evolution (Yoldi-Achalandabaso et al., 2025).

Research on gastrodin in plants, especially regarding the phenolic synthesis pathway, is still relatively limited. In particular, the detailed biosynthetic pathway, regulatory mechanisms, and physiological importance need further confirmation through molecular biology and metabolomics studies. Within the proposed biosynthetic pathway, we have identified potential key regulatory genes: *ADH5* and *ADH11*, and 19 GT genes (*GT3, GT38, GT11, GT16, GT24, GT7, GT39, GT9, GT25, GT41, GT45, GT4, GT8, GT35, GT37, GT46, GT18, GT19* and *GT1*). These genes are hypothesized to play central regulatory roles in the synthesis of phenolic compounds. Glycosylation modifications are highly effective in improving the solubility and enhancing the stability of natural products and are one of the most common and important modifications in nature (Moradi et al., 2016). Glycosidic natural products generated following glycosylation can exhibit significantly enhanced water solubility, pharmacological activity, bioavailability, and thus, higher medicinal value (Ren et al., 2023). The GT family is a large and diverse gene family (Yang et al., 2023). Correlation analysis among 46 GT genes and 6 phenolic compounds revealed significant positive associations between *GT16*, *GT18*, and *GT24* and gastrodin accumulation, suggesting that these genes play key catalytic roles in gastrodin biosynthesis. Several UGTs from groups D and E in phylogenetic analysis can catalyze the conversion of p-hydroxybenzyl alcohol into gastrodin (**Figure 5A**). For instance, UGT73B6 from group D can convert 4-hydroxybenzoic acid into gastrodin in *E. coli*, and an engineered variant, UGT73B6FS, showed more than a 7-fold increase in catalytic efficiency for gastrodin production (Bai et al., 2016). Similarly, AsUGTsyn from group E efficiently catalyzes the transformation of p-hydroxybenzyl alcohol into gastrodin in yeast, achieving a yield of 2.1 g/L (Yin et al., 2022). Additionally, AtUGT from Arabidopsis thaliana demonstrated high efficiency in gastrodin synthesis with a conversion rate of 94.34% (Xia et al., 2024). Based on these findings, we performed protein structure modeling and molecular docking analysis on several GeUGTs from groups D and E, namely GT16, GT17, GT18, GT24, GT42, and GT44 (**Figures 6** and **7**), to predict their potential roles. In GT16, GT17, GT18, and GT44, the catalytic residue His and the key residues within the PSPG box formed interactions with UDPG and p-hydroxybenzyl alcohol. Therefore, we hypothesized that p-hydroxybenzyl alcohol can produce gastrodin under the catalysis of the enzymes encoded by GT16, GT17, GT18, and GT44.

In summary, GR, GB, and GR×GB are the primary commercially circulating varieties in the Chinese market, with distinct appearances and morphological characteristics among different *G. elata* varieties. GR×GB’s superior morphological traits are favored by consumers; however, its content of carbohydrates and phenolic compounds is slightly lower than its parent strains. For medicinal markets and scientific research, traditional GR and GB varieties align more closely with the demands of authentic medicinal material markets and extraction-specific applications due to their higher bioactive compound content. In future studies, we intend to conduct *in vitro* enzymatic assays on GT to fully elucidate the catalytic mechanism underlying gastrodin biosynthesis and enhance the medicinal components in *G. elata*.

## Conclusion

In this study, we systematically analyzed the characteristics of 6 phenolic and 19 sugar metabolic pathways and their molecular regulatory mechanisms in *G. elata* from different origins by integrating metabolomic and transcriptomic analyses. Our results indicate that the accumulation of secondary metabolites (e.g., gastrodin, parishin, p-hydroxybenzyl alcohol, and soluble sugars) was significantly higher in the GR and GB than GR×GB. We identified 96 genes related to the phenolic synthesis pathway, and key functional genes, such as *GT16*, *GT17*, *GT18*, *GT24*, *GT42*, and *GT44*, were analyzed for sequence and structural analyses. Our findings also demonstrated the effects of geographic differences and genetic background on the accumulation of secondary metabolites, providing valuable insights into the metabolites and molecular regulatory mechanisms of *G. elata* metabolites in different varieties from different regions and facilitating further research in this field.

## Data Availability

The RNA-seq data has been submitted to NCBI SRA: PRJNA1249630. Data will be made available on request.
