# Supplementary material for: Integrated transcriptome and metabolome provide insight into phenolics and soluble sugar variation in the different varieties of Gastrodia elata Blume from different areas in China
Source: Front Plant Sci. 2025 Sep 30;16:1656554. doi: 10.3389/fpls.2025.1656554 (PMC12518281; doi:10.3389/fpls.2025.1656554)
Supplement: Supplementary file 1 [file DataSheet1.docx]

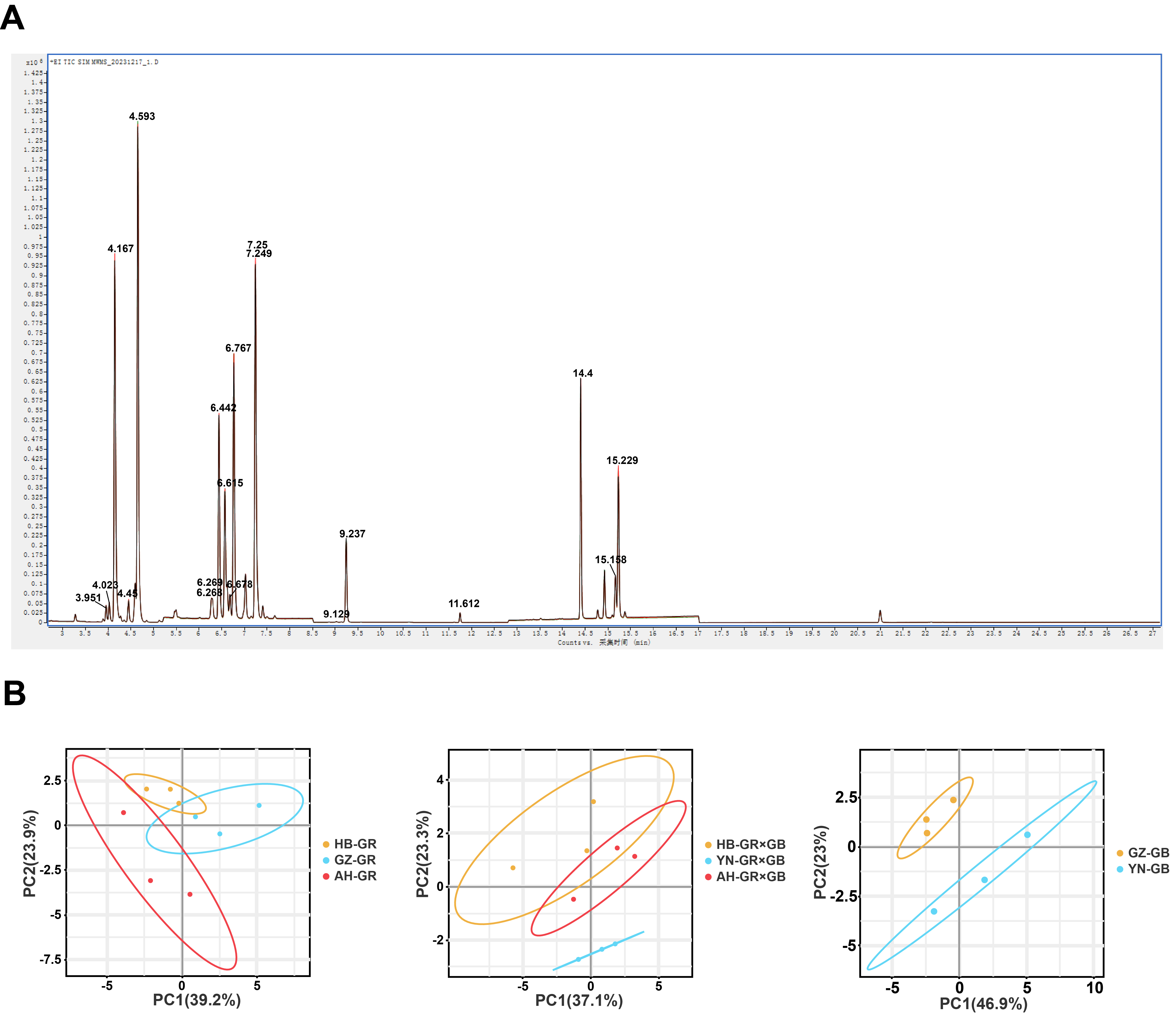


**Fig. S1.** The statistics analysis of *G. elata* soluble sugar metabolites. (A) The total ion overlap diagram. (B) PCA score plot of GR ,GR×GB and GB group.


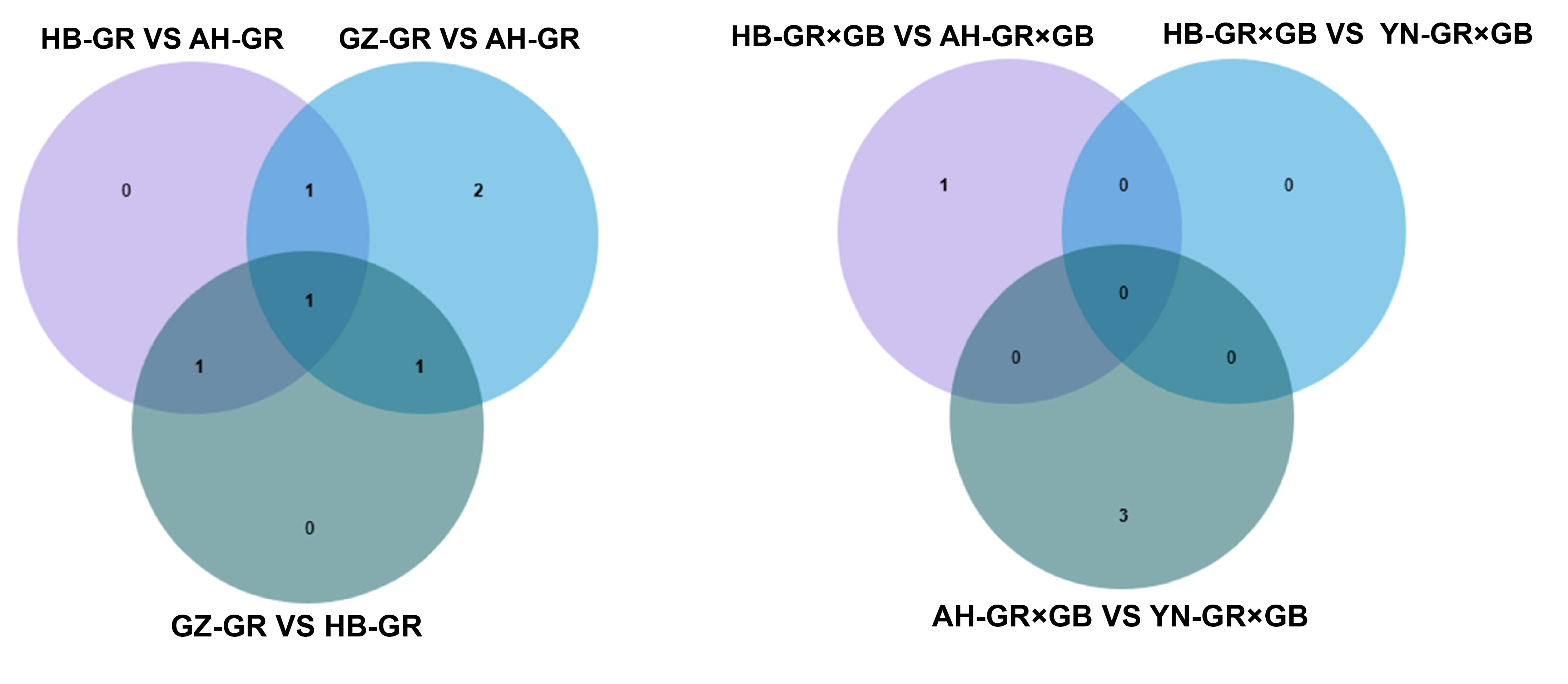


**Fig. S2.** The Venn diagram showed the numbers of the DAMs.

**
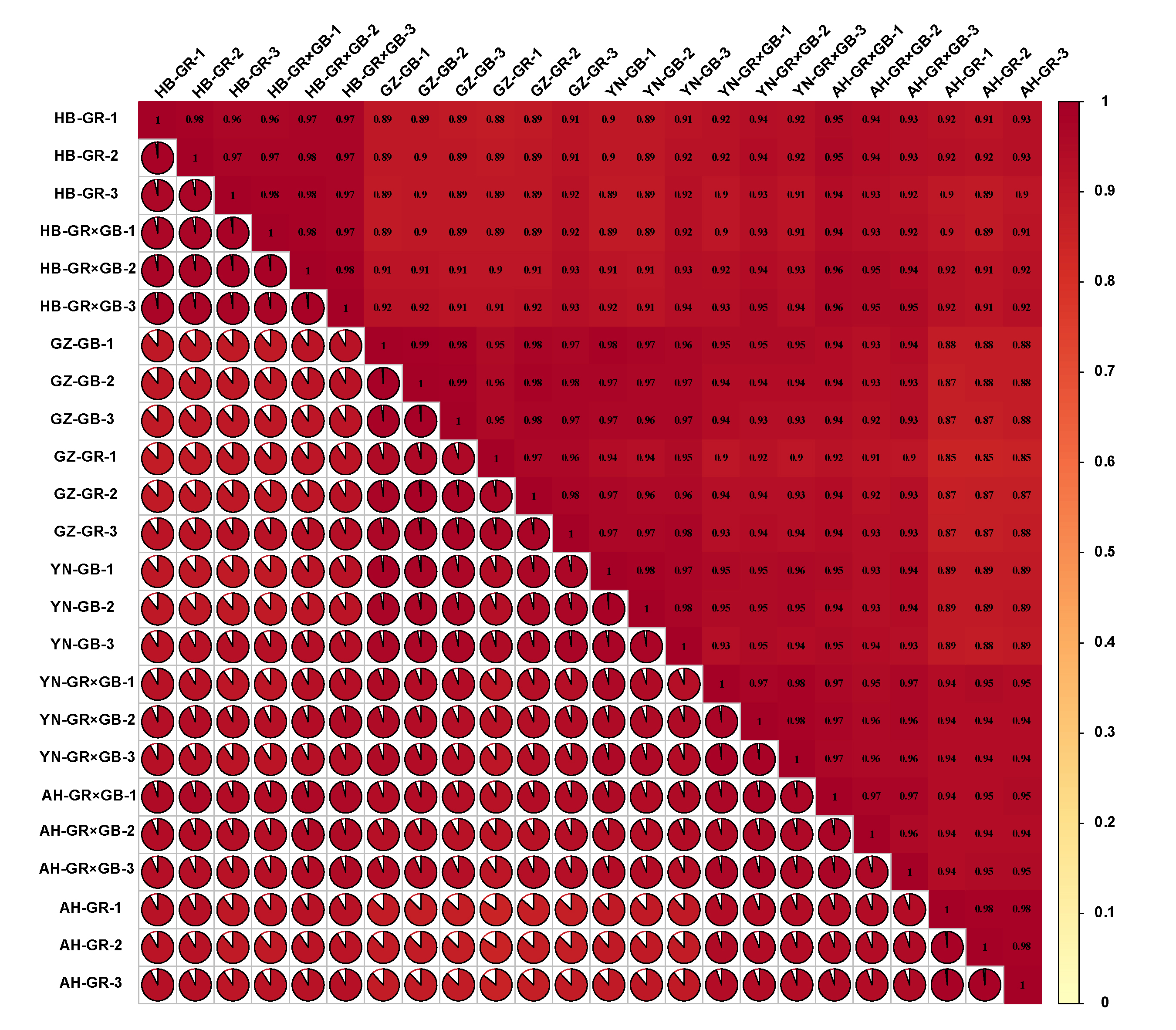
**

**Fig. S3.** The correlation diagram of the transcriptome samples in *G. elata.*

***
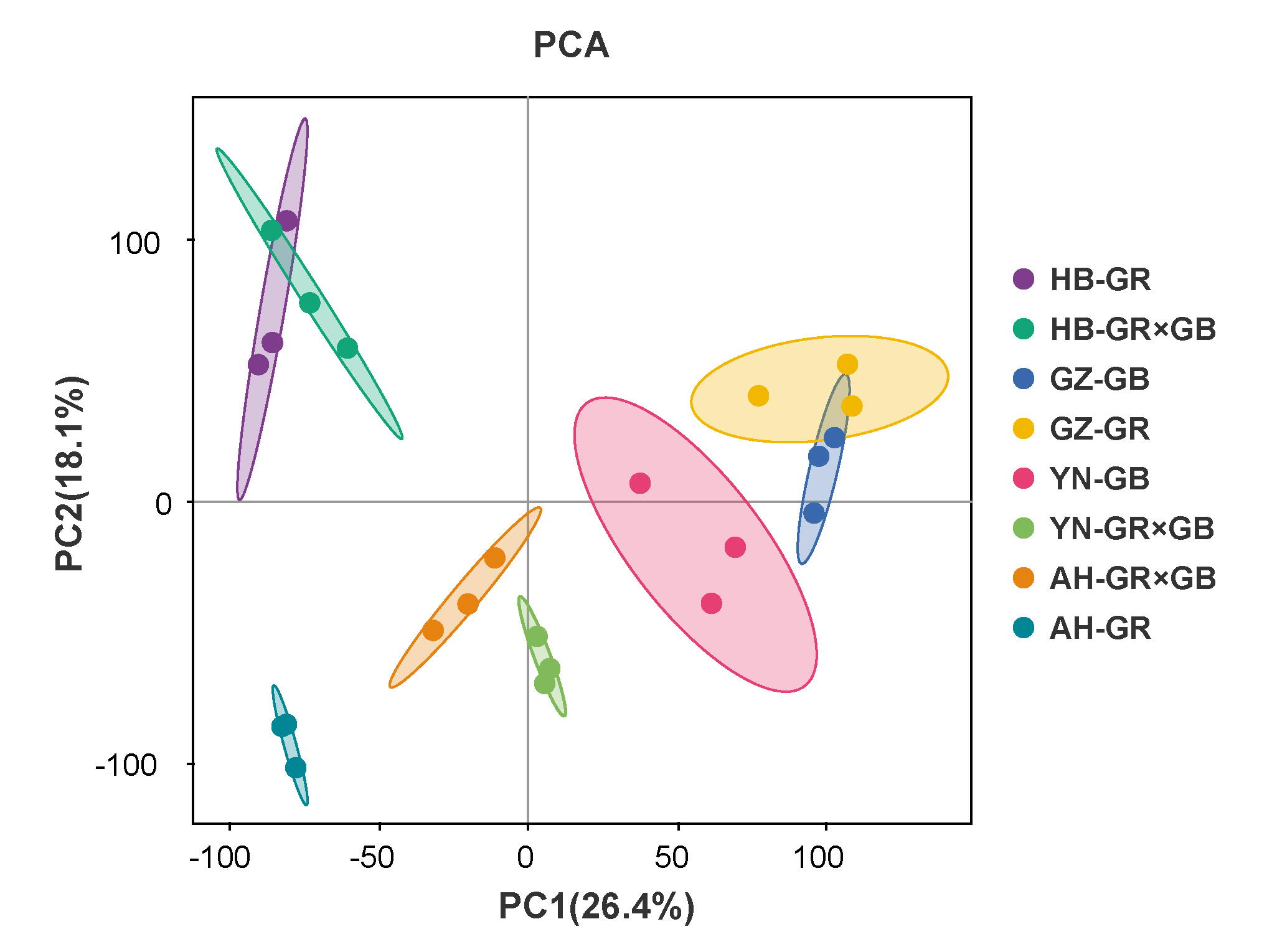
***

**Fig. S4.** PCA score plot of the transcriptome samples in *G. elata.*

*
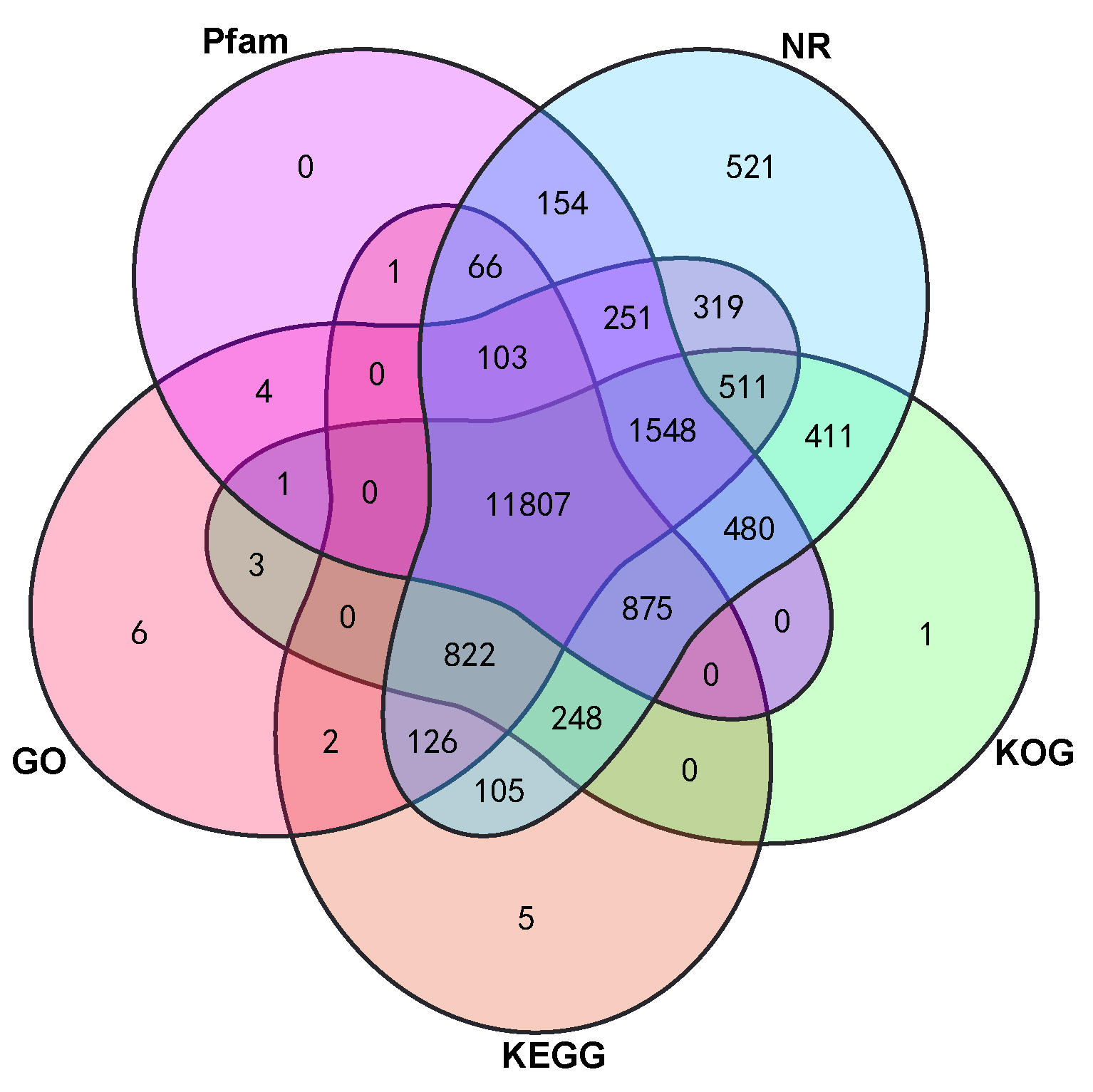
*

**Fig. S5.** Functional annotation results of NR, KOG, Pfam, GO and KEGG.


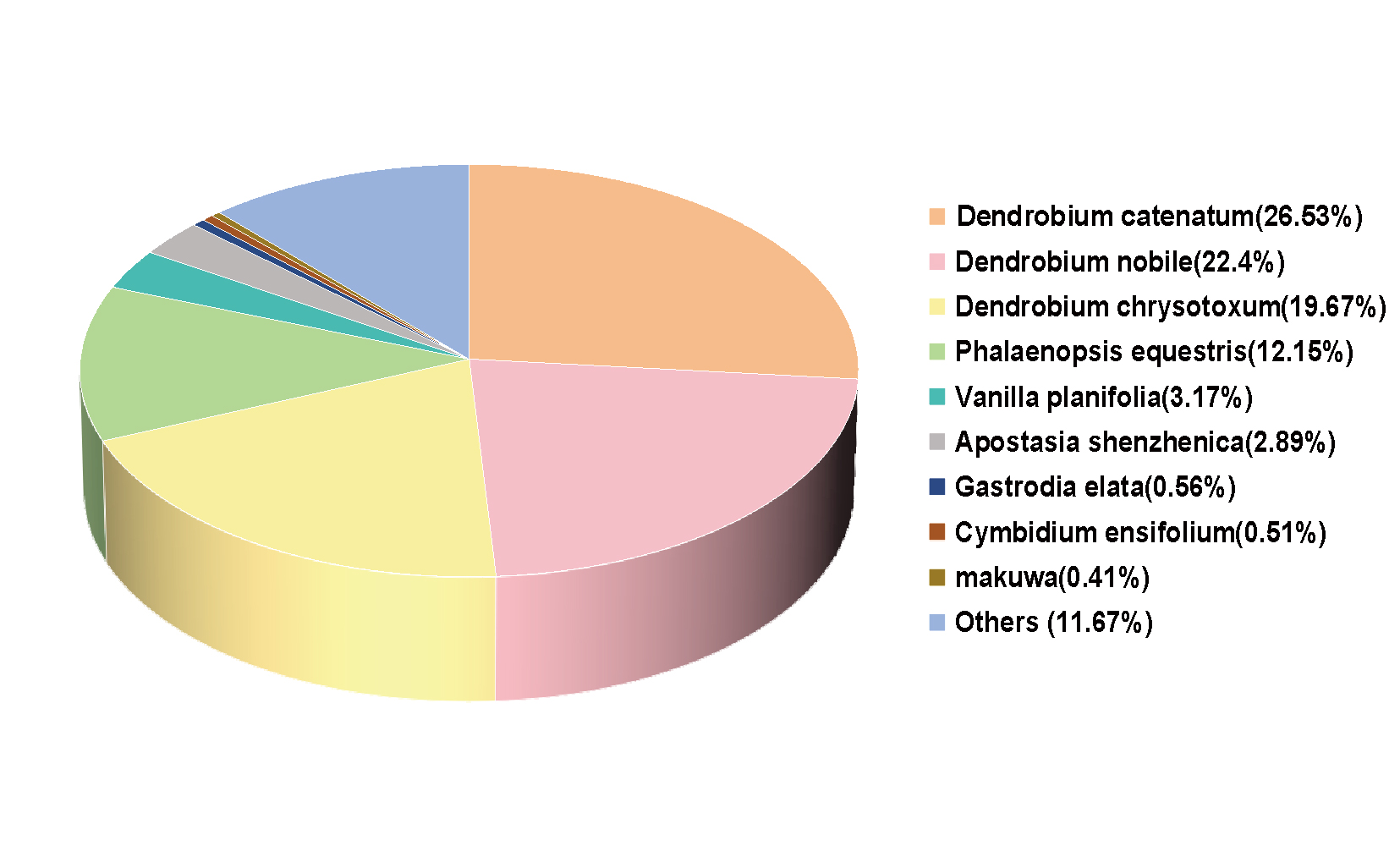


**Fig. S6.** Species distribution of homologous sequences against Nr database.


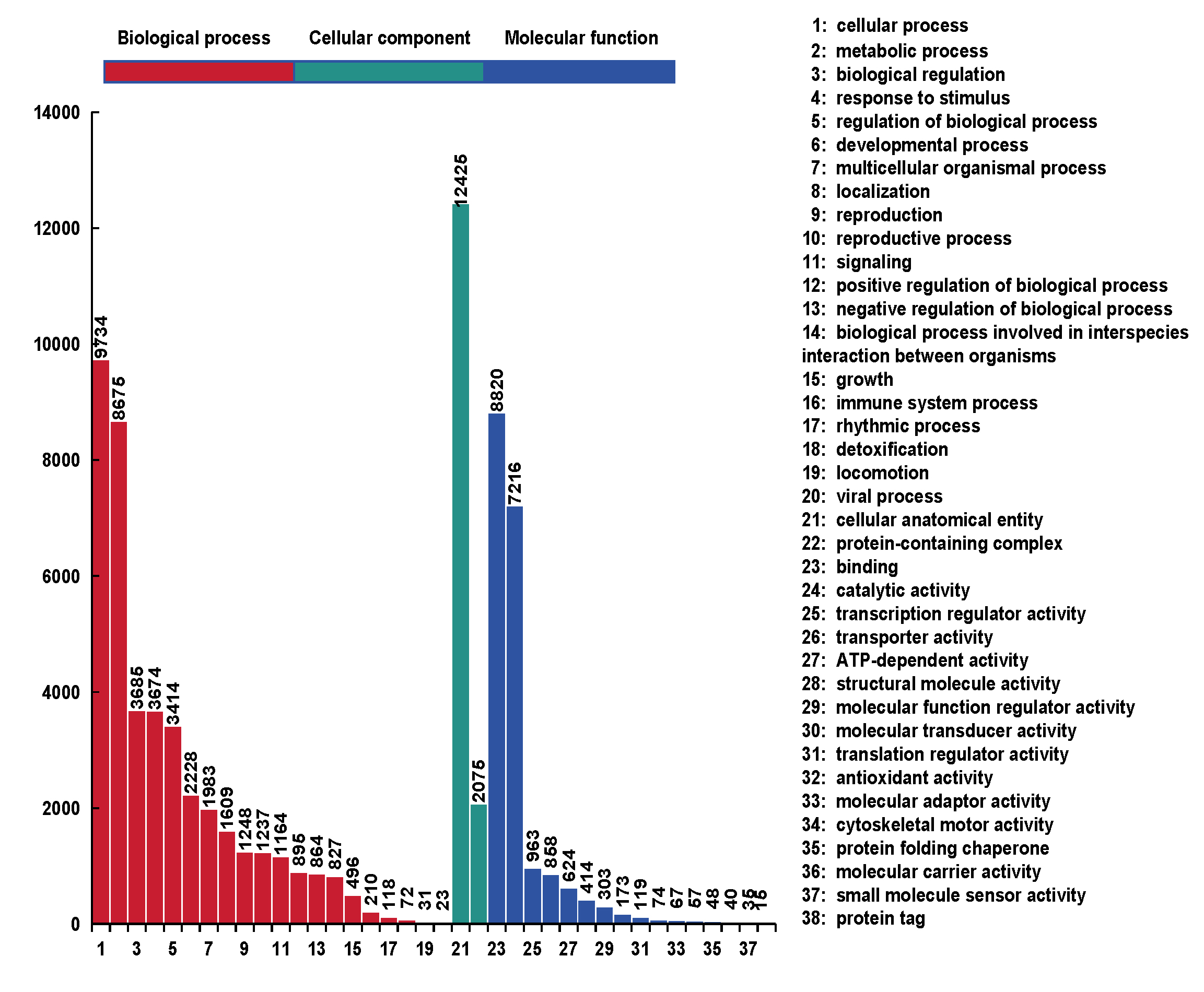


**Fig. S7.** GO functional classification.


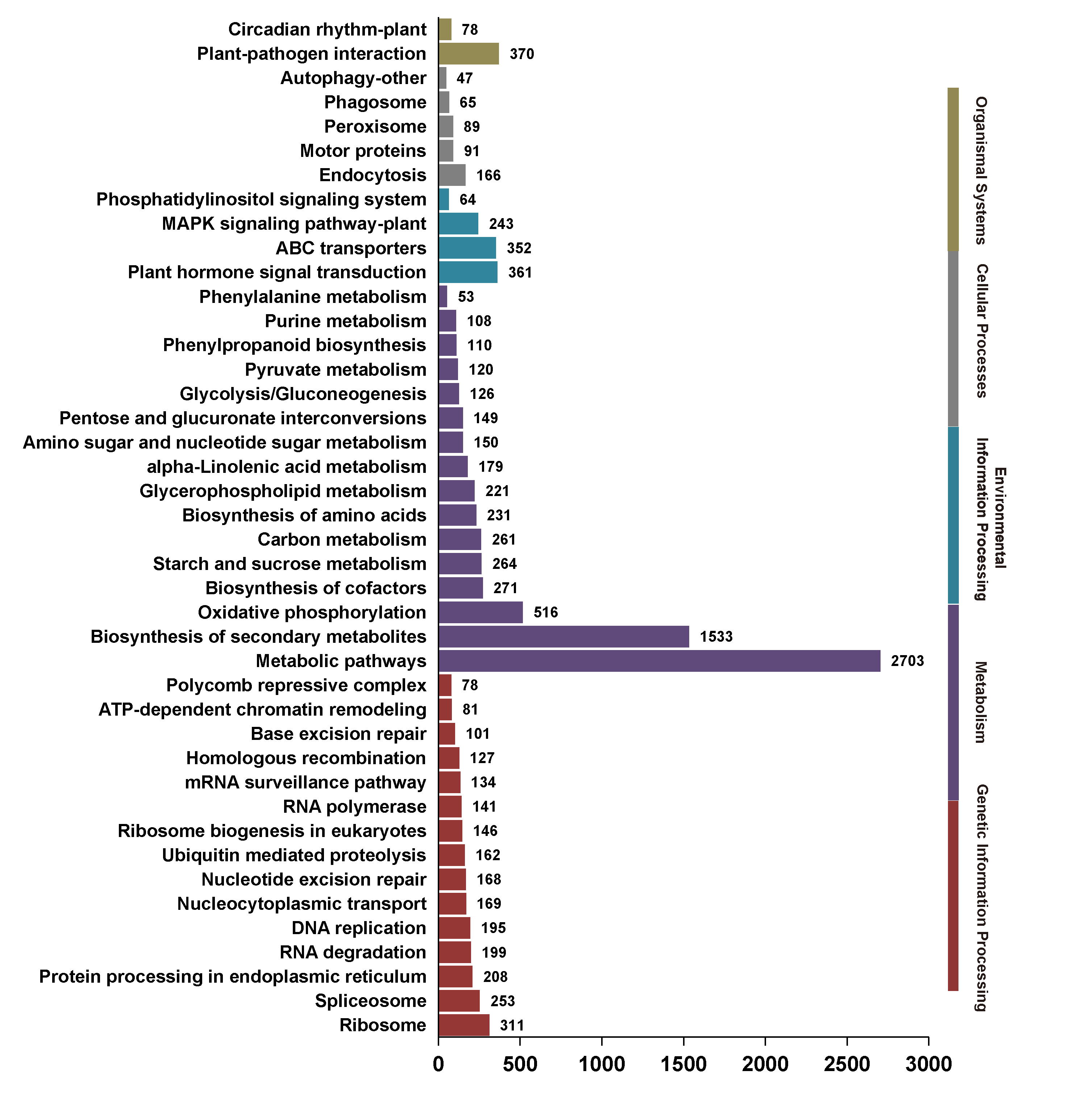


**Fig. S8.** KEGG pathway classification.


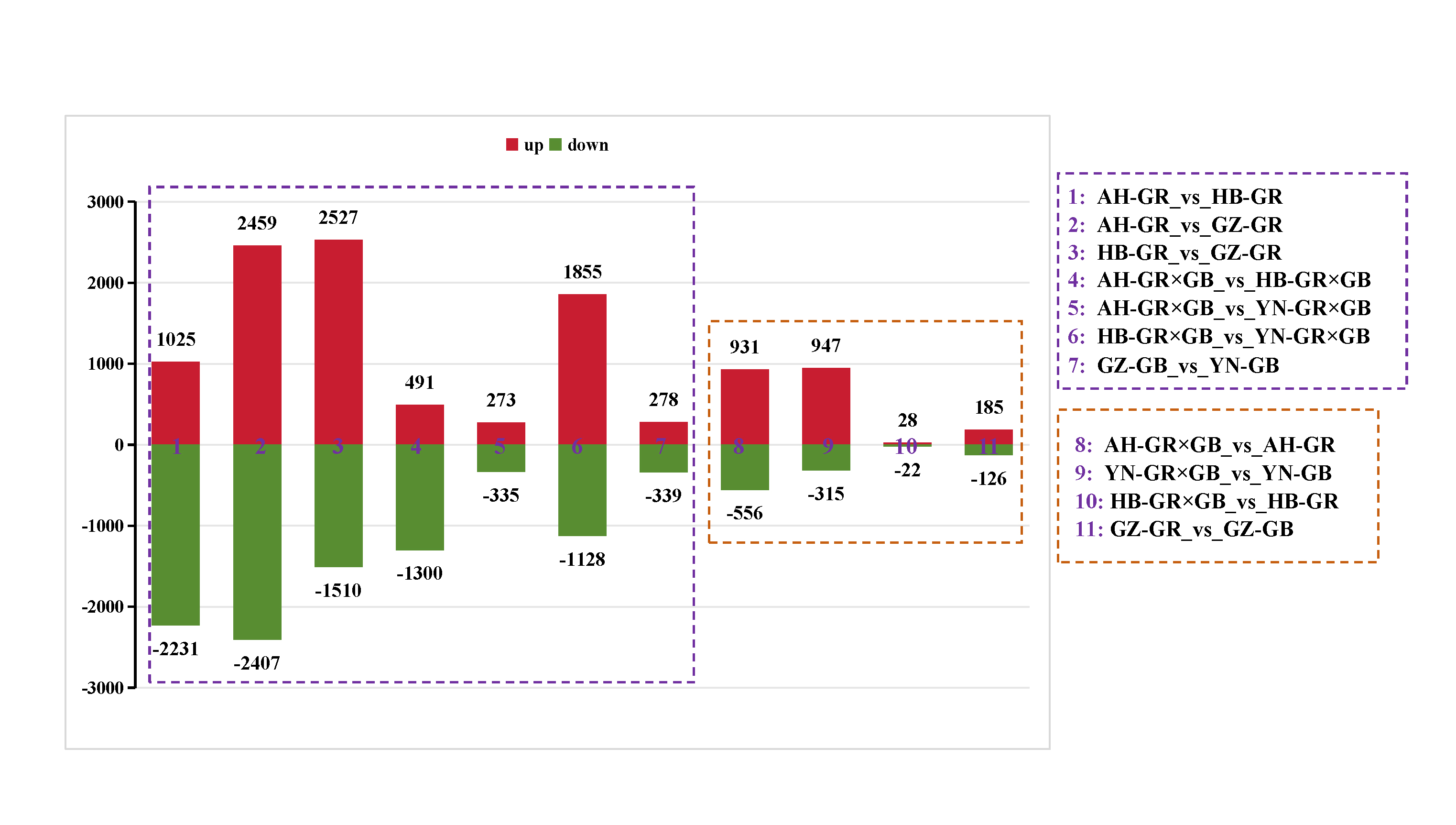


**Fig. S9.** The number of DEGs in *G. elata* of the same variety from different origins and *G. elata* of different varieties from the same origin, red represents up-regulated DEGs, green represents down-regulated DEGs.


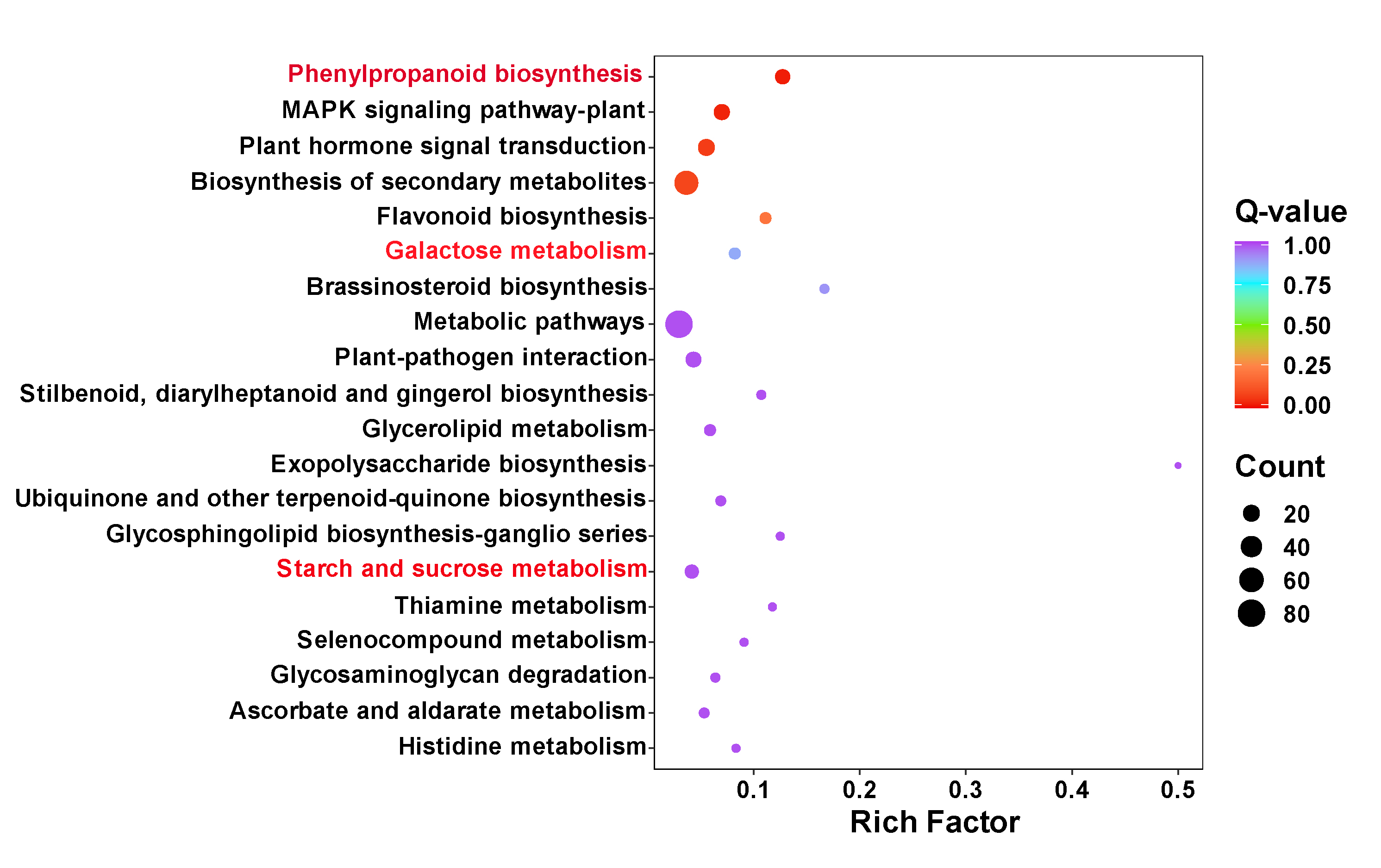


**Fig. S10.** GZ-GB vs YN-GB KEGG Enrichment dotplot.


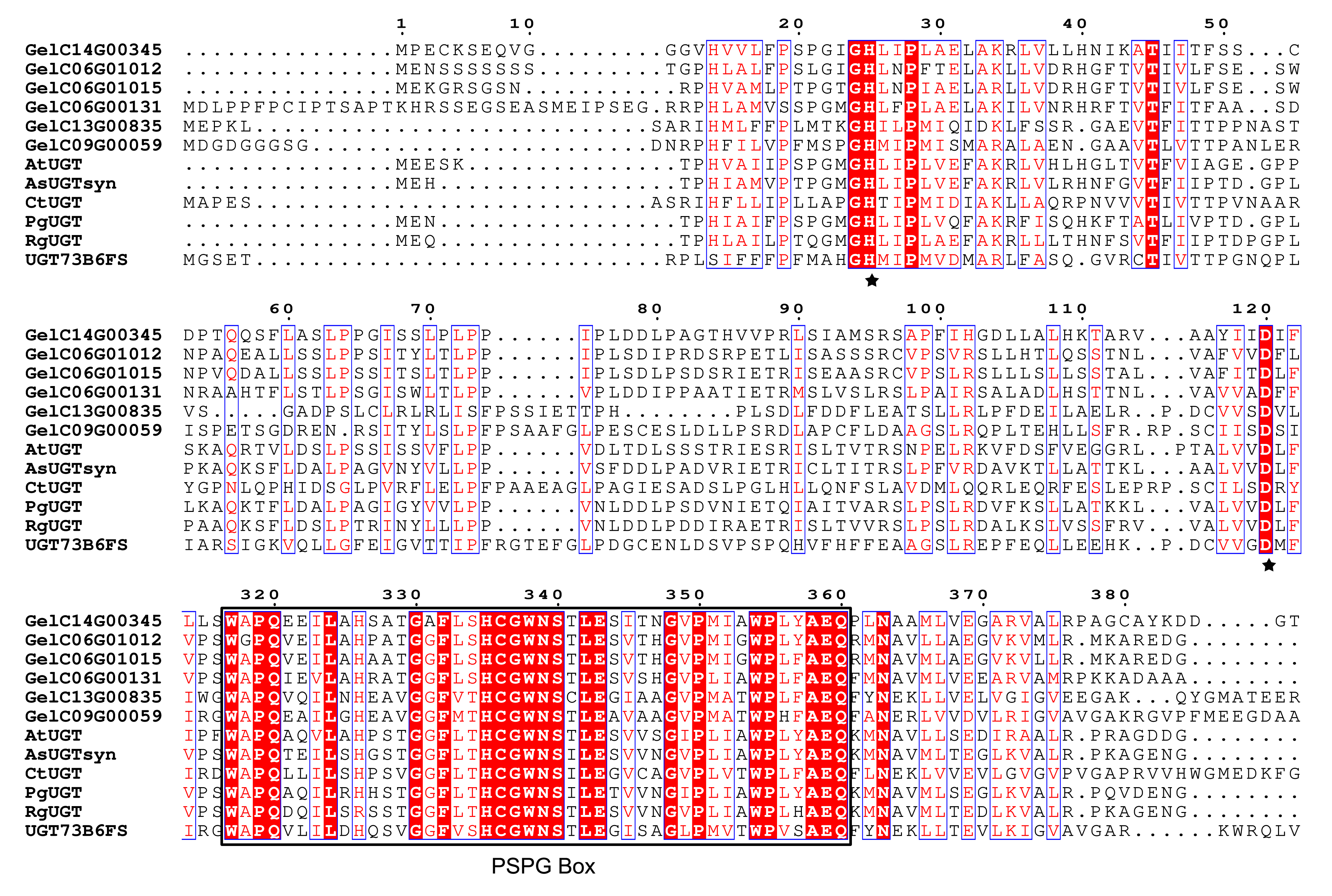


**Fig. S11.** Multiple sequence alignment of GeUGTs proteins in *G. elata*. The black five-pointed stars represent important catalytic residues, black box represents conserved plant secondary product glycosyltransferases (PSPG) box.





**Fig. S12.** The qRT-PCR validation figures of the DEGs in phenolic and soluble sugars biosynthetic pathway.
