# Supplementary material for: Integrated transcriptome and metabolome provide insight into phenolics and soluble sugar variation in the different varieties of Gastrodia elata Blume from different areas in China
Source: Front Plant Sci. 2025 Sep 30;16:1656554. doi: 10.3389/fpls.2025.1656554 (PMC12518281; doi:10.3389/fpls.2025.1656554)
Supplement: Supplementary file 2 [file Table1.docx]

**Table S1.** Material source information of *G. elata*.

| Variety | Abbreviation | Sampling point | Latitude and Longitude | Sample name |
| --- | --- | --- | --- | --- |
| *Gastrodia elata* Bl. f. *elata* | GR | Jinzhai County Anhui Province | AH, 115.51°E, 31.30°N | AH-GR |
|  |  | Luotian County Hubei Province | HB, 115.34°E, 30.59°N | HB-GR |
|  |  | Dejiang County Guizhou Province | GZ, 108.43°E, 28.02°N | GZ-GR |
| *Gastrodia elata* Bl. f. *glauca* S. Chow and *Gastrodia elata* Bl. f. *elata* | GR×GB | Jinzhai County Anhui Province | AH, 115.44°E, 31.33°N | AH-GR×GB |
|  |  | Luotian County Hubei Province | HB, 115.39°E, 30.45°N | HB-GR×GB |
|  |  | Yiliang County Yunnan Province | YN, 104.25°E, 27.47°N | YN-GR×GB |
| *Gastrodia elata* Bl. f. *glauca* S. Chow | GB | Xiuwen County Guizhou Province | GZ, 106.59°E, 26.85°N | GZ-GB |
|  |  | Yiliang County Yunnan Province | YN, 103.67°E, 27.23°N | YN-GB |

**Table S2.** Information of protein molecular docking.

| **Protein** | **UDPG** | | | ***p*- hydroxybenzyl_alcohol** | | | |
| --- | --- | --- | --- | --- | --- | --- | --- |
|  | **Grid center** | **Grid size** | **Binding affinity (kcal/mol)** | **Grid center** | **Grid size** | **Binding affinity (kcal/mol)** | **RMSD**  **（Å）** |
| GT16 | X=25.944 Y=7.604 Z=6.625 | X=78.05 Y=78.05 Z=78.05 | -8.87 | X=25.944 Y=7.604 Z=6.625 | X=79.45 Y=79.45 Z=79.45 | -5.043 | 2.429 |
| GT17 | X=25.944 Y=7.604 Z=6.625 | X=78.75 Y=78.75 Z=78.75 | -8.906 | X=25.944 Y=7.604 Z=6.625 | X=68.95 Y=68.95 Z=68.95 | -5.326 | 2.294 |
| GT18 | X=25.944 Y=7.604 Z=6.625 | X=88.55 Y=88.55 Z=88.55 | -8.308 | X=15.037 Y=35.806 Z=-7.295 | X=76.65 Y=76.65 Z=76.65 | -5.677 | 2.375 |
| GT24 | X=15.037 Y=35.806 Z=-7.295 | X=82.95 Y=82.95 Z=82.95 | -6.199 | X=15.037 Y=35.806 Z=-7.295 | X=82.95 Y=72.4167 Z=82.95 | -5.152 | 2.378 |
| GT42 | X=15.037 Y=35.806 Z=-7.295 | X=68.2833 Y=69.6222 Z=84.35 | -6.524 | X=15.037 Y=35.806 Z=-7.295 | X=68.2833 Y=69.6222 Z=84.35 | -4.375 | 2.340 |
| GT44 | X=16.252 Y=29.765 Z=-5.262 | X=64.4833 Y=76.65 Z=76.65 | -9.68 | X=16.252 Y=29.765 Z=-5.262 | X=64.4833 Y=76.65 Z=76.65 | -4.816 | 2.458 |

**Table S3.** Statistics of molecular docking results.

| **Protein** | **Number of hydrogen bonds** | **Amino acid residues** |
| --- | --- | --- |
| GT16 | 18 | Gly47 His48 Lys274 Ser296 Phe297 Ser299 Gly300 Ser368 Ala370 Gln372 His387 Asn391 Ser392 Glu411 Gln412 |
| GT17 | 13 | Gly22 His25 Ser280 Arg308 Gly351 Gln353 His368 Trp371 Asn372 Ser373 Glu392 Gln393 |
| GT18 | 9 | Phe273 Leu279 Trp345 His363 Trp366 Asn367 Ser368 Glu387 |
| GT24 | 8 | Ile130 Arg204 Arg205 Glu207 Arg208 |
| GT42 | 6 | Typ331 Lys349 Gln350 Tyr351 |
| GT44 | 16 | Gly24 Arg217 Leu244 Ser246 Leu250 Ala318 Gln320 His335 Trp338 Asn339 Ser340 Tyr357 Glu359 Gln360 |

**Table S4.** qRT-PCR Primer.

| **ID** | **Sequence （5’-3’）** |
| --- | --- |
| β-actin-F | GATTCTGGCGACGGAGTTAGT |
| β-actin-R | CTCCAGCTCCTGCTCGTAATC |
| C4H-F | ATTATGTTTGATCGGAGGTTCG |
| C4H-R | CGGAATAAAATCACCGTAGTTG |
| 4CL2-F | CCTCTGAGCAAGCAGTTGATACA |
| 4CL2-R | CACTACTTTGGCTTCCATGTTTG |
| CCoAOMCT1-F | TACTACGAGATCGGGTTACCATT |
| CCoAOMCT1-R | AGTTGCTTCTGAACCAATCTGTC |
| ADH4-F | GGCTGACGCTTTCATCCTG |
| ADH4-R | TGAGCAAGGCCAAGTAAGG |
| GT16-F | GTCGGTTCTGTTCGTGTCCT |
| GT16-R | TGGGCGGTGAAATAGGTAGA |
| GT17-F | TCAGGATCGGAAGAGCGACT |
| GT17-R | CTGCCGAAGGAAACGAAAAT |
| GT18-F | AGACCTGACCTGCGATTTCC |
| GT18-R | GTAGGGGATGGAGGATGTCG |
| UGE1-F | ACCCGGTTGCTTCTCATG |
| UGE1-R | TCGAATCGCGGTTCCATC |
| INV8-F | GAACCATTGCTGCCGTAGAT |
| INV8-R | TCCCACCCCTGAAGAAGAAG |
| GT24-F | GGCGGGGTGTAGGTTTGT |
| GT24-R | TCGAGTTCCAGCCGCAGT |
| GT42-F | CTTTTGTCTGGGCGGTGAG |
| GT42-R | TCGAGGCACGAGTTCCAT |
| GT44-F | GGAAAGGACGAGGGAACG |
| GT44-R | GCAGTGCGACAGGAAAGC |

**Table S5.** The average content of soluble sugars in different *G. elata* (n=3).

| **Compounds(mg/g)** | **AH-GR ± SD** | **HB-GR ± SD** | **GZ-GR ± SD** | **AH-GR×GB ± SD** | **HB-GR×GB**  **± SD** | **YN-GR×GB**  **± SD** | **GZ-GB ± SD** | **YN-GB ± SD** |
| --- | --- | --- | --- | --- | --- | --- | --- | --- |
| Maltose | 0.0793±0.0163 | 0.0743±0.017 | 0.0655±0.0091 | 0.0471±0.0095 | 0.0609±0.0095 | 0.0986±0.0253 | 0.0722±0.0098 | 0.0966±0.0157 |
| Sucrose | 71.3285±17.9908 | 88.8184±17.0085 | 50.9133±6.1092 | 58.0905±8.8528 | 89.7407±22.6608 | 69.103±6.1233 | 64.0141±8.3048 | 56.5355±11.6693 |
| Trehalose | 0.0145±0.0004 | 0.0146±0.0014 | 0.0138±0.001 | 0.0141±0.0022 | 0.0133±0.0008 | 0.0142±0.0019 | 0.0132±0.0007 | 0.0175±0.0029 |
| D-Xylulose | 0.0004±0.0001 | 0.0008±0.0002 | 0.001±0.0003 | 0.0003±0.0001 | 0.0004±0.0001 | 0.0003±0.0002 | 0.0006±0.0002 | 0.0006±0.0001 |
| D-Arabinose | 0.0211±0.0066 | 0.0296±0.0103 | 0.0486±0.0233 | 0.0132±0.0031 | 0.021±0.0039 | 0.0119±0.0014 | 0.0299±0.0032 | 0.0378±0.0038 |
| Xylitol | 0.0005±0.0002 | 0.0031±0.0014 | 0.0084±0.0039 | 0.0054±0.0045 | 0.003±0.0003 | 0.0026±0.0012 | 0.0084±0.0019 | 0.0055±0.0031 |
| D-Sorbitol | 0.0056±0.003 | 0.0032±0.0011 | 0.0039±0.0005 | 0.0071±0.0044 | 0.0033±0.0005 | 0.0042±0.001 | 0.006±0.0015 | 0.0063±0.0026 |
| L-Rhamnose | 0.0045±0.0002 | 0.0053±0.0003 | 0.0048±0.0008 | 0.0044±0.0003 | 0.0052±0.0005 | 0.0037±0.0003 | 0.0054±0.0004 | 0.0043±0.0002 |
| D-Mannose | 0.0625±0.0406 | 0.0775±0.0278 | 0.1523±0.0415 | 0.044±0.0178 | 0.0471±0.029 | 0.039±0.0054 | 0.1447±0.0485 | 0.1926±0.0884 |
| Inositol | 0.1075±0.0269 | 0.0584±0.0134 | 0.0561±0.014 | 0.0372±0.0099 | 0.038±0.011 | 0.0198±0.0048 | 0.0149±0.0015 | 0.0318±0.0165 |
| D-Glucuronic acid | 0.0327±0.0128 | 0.0139±0.0025 | 0.0398±0.0535 | 0.0106±0.0009 | 0.0119±0.0033 | 0.009±0.0005 | 0.0114±0.0012 | 0.009±0.0008 |
| Glucose | 18.7861±10.3969 | 10.5116±5.0255 | 18.3444±10.2232 | 2.6802±0.6389 | 4.8462±3.3183 | 6.5643±1.8029 | 10.9797±5.9292 | 23.9854±10.5297 |
| D-Galactose | 0.0911±0.072 | 0.1109±0.0363 | 0.1736±0.0302 | 0.0446±0.0216 | 0.0579±0.0348 | 0.0569±0.0109 | 0.1471±0.067 | 0.2372±0.1032 |
| D-Fructose | 20.6806±14.1256 | 13.2837±6.6417 | 18.1608±5.6819 | 3.4699±2.0636 | 5.2008±2.7987 | 5.2737±2.1848 | 11.4975±4.0563 | 23.8357±7.3863 |
| 1,5-Anhydroglucitol | 0.1058±0.0261 | 0.1334±0.0294 | 0.07±0.0069 | 0.0663±0.0209 | 0.0855±0.0117 | 0.0575±0.0037 | 0.0652±0.004 | 0.0674±0.0033 |
| D-Xylose | 0.2997±0.1667 | 0.4828±0.237 | 0.496±0.1757 | 0.0935±0.0515 | 0.17±0.034 | 0.1485±0.0378 | 0.3861±0.0152 | 0.358±0.0791 |
| 2-Acetamido-2-deoxy-D-glucopyranose | 0±0 | 0±0 | 0.0779±0.0202 | 0±0 | 0±0 | 0±0 | 0.0232±0.0403 | 0.0217±0.0306 |
| Methyl beta-D-galactopyranoside | 0.0108±0.0015 | 0.0086±0.0009 | 0.0109±0.001 | 0.0115±0.0014 | 0.0089±0.0019 | 0.0121±0.0003 | 0.009±0.0008 | 0.0132±0.0009 |
| D-Mannose-6-phosphate sodium salt | 0.0755±0.0129 | 0.0592±0.0034 | 0.055±0.0008 | 0.0609±0.007 | 0.065±0.013 | 0.055±0.0057 | 0.0615±0.0076 | 0.0624±0.0061 |

**Table S6.** The differential soluble sugars metabolites of results.

| **Index** | **Compounds** | **Class** | **Pvalue** | **Fold**  **Change** | **Log_2_FC** | **Type** | **Group** |
| --- | --- | --- | --- | --- | --- | --- | --- |
| Xylulose | D-Xylulose | monosaccharide | 0.0659 | 2.0291 | 1.0208 | up | HB-GR vs AH-GR |
| Xylitol | Xylitol | monosaccharide | 0.0845 | 6.2238 | 2.6378 | up | HB-GR vs AH-GR |
| Glucuronic-A | D-Glucuronic acid | monosaccharide | 0.1227 | 0.4271 | -1.2273 | down | HB-GR vs AH-GR |
| 2-Ace-2-Deo-D-Glucosamine | 2-Acetamido-2-deoxy-D-glucopyranose | monosaccharide | 0.0217 | Inf | Inf | up | GZ-GR vs AH-GR |
| Xylulose | D-Xylulose | monosaccharide | 0.0362 | 2.7940 | 1.4823 | up | GZ-GR vs AH-GR |
| D-Ara | D-Arabinose | monosaccharide | 0.1695 | 2.3056 | 1.2052 | up | GZ-GR vs AH-GR |
| Xylitol | Xylitol | monosaccharide | 0.0708 | 17.0733 | 4.0937 | up | GZ-GR vs AH-GR |
| Man | D-Mannose | monosaccharide | 0.0553 | 2.4376 | 1.2854 | up | GZ-GR vs AH-GR |
| 2-Ace-2-Deo-D-Glucosamine | 2-Acetamido-2-deoxy-D-glucopyranose | monosaccharide | 0.0217 | 0.0000 | -Inf | down | HB-GR vs GZ-GR |
| Xylitol | Xylitol | monosaccharide | 0.1268 | 0.3645 | -1.4559 | down | HB-GR vs GZ-GR |
| Glucuronic-A | D-Glucuronic acid | monosaccharide | 0.4910 | 0.3506 | -1.5119 | down | HB-GR vs GZ-GR |
| Sorbitol | D-Sorbitol | monosaccharide | 0.2717 | 2.1405 | 1.0979 | up | AH-GR×GB vs HB-GR×GB |
| Mal | Maltose | disaccharide | 0.0580 | 2.0927 | 1.0654 | up | YN-GR×GB vs AH-GR×GB |
| Xylitol | Xylitol | monosaccharide | 0.4042 | 0.4868 | -1.0387 | down | YN-GR×GB vs AH-GR×GB |
| Glu | Glucose | monosaccharide | 0.0520 | 2.4492 | 1.2923 | up | YN-GR×GB vs AH-GR×GB |
| Inositol | Inositol | monosaccharide | 0.2826 | 2.1385 | 1.0966 | up | YN-GB vs GZ-GB |
| Glu | Glucose | monosaccharide | 0.2168 | 2.1845 | 1.1273 | up | YN-GB vs GZ-GB |
| Fru | D-Fructose | monosaccharide | 0.1274 | 2.0731 | 1.0518 | up | YN-GB vs GZ-GB |
| 2-Ace-2-Deo-D-Glucosamine | 2-Acetamido-2-deoxy-D-glucopyranose | monosaccharide | 0.1281 | 3.3501 | 1.7442 | up | GZ-GR vs GZ-GB |
| Inositol | Inositol | monosaccharide | 0.0350 | 3.7707 | 1.9148 | up | GZ-GR vs GZ-GB |
| Glucuronic-A | D-Glucuronic acid | monosaccharide | 0.4549 | 3.4960 | 1.8057 | up | GZ-GR vs GZ-GB |
| Glu | Glucose | monosaccharide | 0.1894 | 0.4610 | -1.1171 | down | HB-GR×GB vs HB-GR |
| Fru | D-Fructose | monosaccharide | 0.1579 | 0.3915 | -1.3528 | down | HB-GR×GB vs HB-GR |
| Xylose | D-Xylose | monosaccharide | 0.1470 | 0.3521 | -1.5061 | down | HB-GR×GB vs HB-GR |
| Xylitol | Xylitol | monosaccharide | 0.2029 | 10.9111 | 3.4477 | up | AH-GR×GB vs AH-GR |
| Inositol | Inositol | monosaccharide | 0.0333 | 0.3466 | -1.5287 | down | AH-GR×GB vs AH-GR |
| Glucuronic-A | D-Glucuronic acid | monosaccharide | 0.0965 | 0.3261 | -1.6166 | down | AH-GR×GB vs AH-GR |
| Glu | Glucose | monosaccharide | 0.1148 | 0.1427 | -2.8093 | down | AH-GR×GB vs AH-GR |
| Gal | D-Galactose | monosaccharide | 0.3808 | 0.4889 | -1.0324 | down | AH-GR×GB vs AH-GR |
| Fru | D-Fructose | monosaccharide | 0.1668 | 0.1678 | -2.5753 | down | AH-GR×GB vs AH-GR |
| Xylose | D-Xylose | monosaccharide | 0.1565 | 0.3119 | -1.6806 | down | AH-GR×GB vs AH-GR |
| Xylulose | D-Xylulose | monosaccharide | 0.1042 | 0.4218 | -1.2452 | down | YN-GR×GB vs YN-GB |
| D-Ara | D-Arabinose | monosaccharide | 0.0068 | 0.3151 | -1.6661 | down | YN-GR×GB vs YN-GB |
| Xylitol | Xylitol | monosaccharide | 0.3166 | 0.4727 | -1.0810 | down | YN-GR×GB vs YN-GB |
| Man | D-Mannose | monosaccharide | 0.1328 | 0.2023 | -2.3056 | down | YN-GR×GB vs YN-GB |
| Glu | Glucose | monosaccharide | 0.1417 | 0.2737 | -1.8694 | down | YN-GR×GB vs YN-GB |
| Gal | D-Galactose | monosaccharide | 0.1311 | 0.2400 | -2.0589 | down | YN-GR×GB vs YN-GB |
| Fru | D-Fructose | monosaccharide | 0.0636 | 0.2213 | -2.1762 | down | YN-GR×GB vs YN-GB |
| Xylose | D-Xylose | monosaccharide | 0.0497 | 0.4149 | -1.2691 | down | YN-GR×GB vs YN-GB |

**Table S7.** The average content of phenolics in different *G. elata* (n=3).

| **Compounds** | **AH-GR ± SD** | **HB-GR ± SD** | **GZ-GR ± SD** | **AH-GR×GB**  **± SD** | **HB-GR×GB**  **± SD** | **YN-GR×GB**  **± SD** | **GZ-GB ± SD** | **YN-GB ± SD** |
| --- | --- | --- | --- | --- | --- | --- | --- | --- |
| Gastrodin | 6.6977±1.1871 | 2.3182±0.6981 | 7.5485±3.0921 | 2.4287±0.2898 | 2.2604±0.1382 | 2.2847±0.9374 | 3.55±0.2435 | 3.6057±0.6179 |
| p-Hydroxybenzyl Alcohol | 0.9856±0.1221 | 0.6339±0.059 | 0.2418±0.1498 | 0.3548±0.0824 | 0.4042±0.1164 | 0.462±0.1412 | 0.2234±0.189 | 2.2098±0.34 |
| Parishin A | 8.8847±1.9934 | 10.1076±3.7238 | 17.2269±8.1407 | 6.3326±1.7195 | 10.227±2.5511 | 4.9487±3.445 | 11.9121±1.6442 | 8.7585±2.9472 |
| Parishin B | 7.8399±0.8094 | 7.6354±1.9908 | 9.9866±1.8664 | 6.3747±0.6948 | 7.9356±0.6086 | 6.6644±2.8873 | 10.6685±2.0388 | 11.369±1.3359 |
| Parishin C | 1.2082±0.2173 | 0.7864±0.2552 | 2.1294±0.6884 | 0.8858±0.1877 | 0.8209±0.126 | 0.8572±0.4857 | 1.6225±0.2793 | 1.6246±0.3763 |
| Parishin E | 4.7458±0.9521 | 10.9723±0.9355 | 9.2285±4.3817 | 10.8146±2.5991 | 12.9972±1.5847 | 6.3697±1.6278 | 12.0235±1.423 | 7.9224±1.9721 |
| Total | 30.362±2.6559 | 32.4539±6.0648 | 46.3616±17.6264 | 27.1912±3.034 | 34.6453±4.8621 | 21.5866±9.2777 | 40±5.5831 | 35.49±3.7059 |

**Table S8.** Summary of RNA-seq results in *G. elata*.

| **Sample** | **Raw Reads** | **Clean Reads** | **Error Rate(%)** | **Q20(%)** | **Q30(%)** | **GC Content(%)** | **Mapped reads** | **Mapped ratio (%)** |
| --- | --- | --- | --- | --- | --- | --- | --- | --- |
| HB-GR-1 | 50472522 | 48957336 | 0.03 | 97.49 | 93.29 | 46.02 | 46304564 | 94.58 |
| HB-GR-2 | 48863524 | 47474828 | 0.03 | 97.46 | 93.15 | 46.47 | 45023919 | 94.84 |
| HB-GR-3 | 55257036 | 53899406 | 0.03 | 97.55 | 93.27 | 46.12 | 51547622 | 95.64 |
| HB-GR×GB-1 | 47631552 | 46114378 | 0.03 | 97.35 | 92.95 | 46.2 | 43823257 | 95.03 |
| HB-GR×GB-2 | 55488922 | 54050494 | 0.03 | 97.44 | 93.1 | 46.13 | 51559247 | 95.39 |
| HB-GR×GB-3 | 51921678 | 50406738 | 0.03 | 97.4 | 93.04 | 46.13 | 48012877 | 95.25 |
| GZ-GB-1 | 49816868 | 48668566 | 0.03 | 97.38 | 92.94 | 46.89 | 49696802 | 97.57 |
| GZ-GB-2 | 44583886 | 43248992 | 0.03 | 97.64 | 93.52 | 46.19 | 54333154 | 97.36 |
| GZ-GB-3 | 47405518 | 45964538 | 0.03 | 97.35 | 92.83 | 45.43 | 48990425 | 96.92 |
| GZ-GR-1 | 53043230 | 50935924 | 0.03 | 97.79 | 93.84 | 47.11 | 47359378 | 97.31 |
| GZ-GR-2 | 57443220 | 55808458 | 0.03 | 97.47 | 93.12 | 46.56 | 42187184 | 97.54 |
| GZ-GR-3 | 52308138 | 50545506 | 0.03 | 97.2 | 92.54 | 45.92 | 44735937 | 97.33 |
| YN-GB-1 | 54170720 | 52519490 | 0.03 | 97.66 | 93.6 | 47.05 | 51047426 | 97.20 |
| YN-GB-2 | 49807884 | 48411318 | 0.03 | 97.66 | 93.51 | 46.5 | 47131637 | 97.36 |
| YN-GB-3 | 58076648 | 56588612 | 0.03 | 97.49 | 93.19 | 46.45 | 55052695 | 97.29 |
| YN-GR×GB-1 | 51102606 | 49600812 | 0.03 | 97.55 | 93.3 | 45.86 | 47136139 | 95.03 |
| YN-GR×GB-2 | 58330800 | 56780560 | 0.03 | 97.6 | 93.43 | 46.64 | 53916375 | 94.96 |
| YN-GR×GB-3 | 51439480 | 49880374 | 0.03 | 97.62 | 93.5 | 46.68 | 47244644 | 94.72 |
| AH-GR×GB-1 | 46398048 | 44572824 | 0.03 | 97.27 | 92.69 | 46.78 | 42117516 | 94.49 |
| AH-GR×GB-2 | 45887566 | 43281054 | 0.03 | 97.87 | 94.12 | 47.61 | 40944132 | 94.60 |
| AH-GR×GB-3 | 46283988 | 44306314 | 0.03 | 97.29 | 92.85 | 46.39 | 42235524 | 95.33 |
| AH-GR-1 | 51530864 | 48146244 | 0.03 | 97.7 | 93.74 | 46.96 | 44586066 | 92.61 |
| AH-GR-2 | 58014800 | 55779050 | 0.03 | 97.27 | 92.78 | 46.52 | 51515487 | 92.36 |
| AH-GR-3 | 46821898 | 44623150 | 0.03 | 97.21 | 92.61 | 46.31 | 41029087 | 91.95 |

**Table S9.** Transcriptome sequence function annotation table.

| **Annotated Database** | **GO** | **KEGG** | **KOG** | **Pfam** | **Swiss-Prot** | **Trembl** | **NR** | **All** |
| --- | --- | --- | --- | --- | --- | --- | --- | --- |
| **Annotated Number** | 15,503 | 14,160 | 11,452 | 15,290 | 13,109 | 18,278 | 18,347 | 18,415 |
| **Annotated Percent (%)** | 66.93 | 61.13 | 49.44 | 66.01 | 56.60 | 78.91 | 79.21 | 79.51 |

**Table S10.** Number of genes encoding key enzymes.

| **Enzyme** | **Abbreviations** | **EC number** | **ID** | **Rename** |
| --- | --- | --- | --- | --- |
| Phenylalanine ammonialyase | PAL | 4.3.1.24 | GelC02G01892 | PAL1 |
|  |  |  | GelC02G01897 | PAL2 |
|  |  |  | GelC02G01898 | PAL3 |
|  |  |  | GelC03G00168 | PAL4 |
| Cinnamate4-hydroxylase | C4H | 1.14.14.91 | GelC02G00339 | C4H |
| 4-comarate coenzymeA ligase | 4CL | 6.2.1.12, 6.2.1.- | GelC01G00992 | 4CL1 |
|  |  |  | GelC03G01279 | 4CL2 |
|  |  |  | GelC03G01284 | 4CL3 |
|  |  |  | GelC04G00359 | 4CL4 |
|  |  |  | GelC04G00787 | 4CL5 |
|  |  |  | GelC04G01345 | 4CL6 |
|  |  |  | GelC05G00275 | 4CL7 |
|  |  |  | GelC11G00236 | 4CL8 |
|  |  |  | GelC11G00237 | 4CL9 |
|  |  |  | GelC15G00807 | 4CL10 |
| Shikimate O-hydroxycinnamoyltransferase | HCT | 2.3.1.133 | GelC01G01170 | HCT1 |
|  |  |  | GelC02G01633 | HCT2 |
|  |  |  | GelC05G00574 | HCT3 |
|  |  |  | GelC09G00015 | HCT4 |
|  |  |  | GelC17G00246 | HCT5 |
| 5-O-(4-coumaroyl)-D-quinate3'-monooxygenase | C3H | 1.14.14.96 | GelC04G00386 | C3H1 |
|  |  |  | GelC10G00895 | C3H2 |
| Caffeoyl-CoA O-methyltransferase | CCoAMT | 2.1.1.104 | GelC01G00401 | CCoAOMT1 |
|  |  |  | GelC05G01100 | CCoAOMT2 |
|  |  |  | GelC05G01104 | CCoAOMT3 |
|  |  |  | GelC05G01102 | CCoAOMT4 |
| Alcoholdehydrogenase | ADH | 1.1.1.195 | GelC02G00646 | ADH1 |
|  |  |  | GelC02G01650 | ADH2 |
|  |  |  | GelC03G01449 | ADH3 |
|  |  |  | GelC07G00442 | ADH4 |
|  |  |  | GelC11G00635 | ADH5 |
|  |  |  | GelC12G00835 | ADH6 |
|  |  |  | GelC15G00204 | ADH7 |
|  |  |  | GelC15G00347 | ADH8 |
|  |  |  | GelC15G00421 | ADH9 |
|  |  |  | GelC15G00516 | ADH10 |
|  |  |  | GelC16G00621 | ADH11 |
| Pyruvatedehydrogenase/pyruvate dehydrogenase complex | aceE | 1.2.4.1 | GelC02G01459 | aceE1 |
|  |  |  | GelC02G01800 | aceE2 |
|  |  |  | GelC02G01801 | aceE3 |
|  |  |  | GelC06G00810 | aceE4 |
|  |  |  | GelC07G00516 | aceE5 |
|  |  |  | GelC12G01017 | aceE6 |
|  |  |  | GelC16G00576 | aceE7 |
| Dihydrolipoyllysine-residue acetyltransferase | DLAT | 2.3.1.12 | GelC07G00880 | DLAT1 |
|  |  |  | GelC08G00152 | DLAT2 |
|  |  |  | GelC08G00603 | DLAT3 |
|  |  |  | GelC09G00565 | DLAT4 |
| Citrate symthase | CS | 2.3.3.1 | GelC04G00926 | CS1 |
|  |  |  | GelC12G00898 | CS2 |
| Glucosyl transferase | GT | 2.4.x.y | GelC01G01713 | GT1 |
|  |  |  | GelC01G01729 | GT2 |
|  |  |  | GelC03G00779 | GT3 |
|  |  |  | GelC03G00917 | GT4 |
|  |  |  | GelC04G00563 | GT5 |
|  |  |  | GelC04G01317 | GT6 |
|  |  |  | GelC04G01503 | GT7 |
|  |  |  | GelC05G00238 | GT8 |
|  |  |  | GelC05G00272 | GT9 |
|  |  |  | GelC05G00499 | GT10 |

| **Enzyme** | **Abbreviations** | **ECnumber** | **ID** | **Rename** |
| --- | --- | --- | --- | --- |
| Glucosyl transferase | GT | 2.4.x.y | GelC05G00996 | GT13 |
|  |  |  | GelC05G01145 | GT14 |
|  |  |  | GelC05G01148 | GT15 |
|  |  |  | GelC06G00131 | GT16 |
|  |  |  | GelC06G01012 | GT17 |
|  |  |  | GelC06G01015 | GT18 |
|  |  |  | GelC07G00061 | GT19 |
|  |  |  | GelC07G00470 | GT20 |
|  |  |  | GelC07G00929 | GT21 |
|  |  |  | GelC08G00814 | GT23 |
|  |  |  | GelC09G00059 | GT24 |
|  |  |  | GelC09G00175 | GT25 |
|  |  |  | GelC09G00739 | GT26 |
|  |  |  | GelC09G00742 | GT27 |
|  |  |  | GelC09G01156 | GT28 |
|  |  |  | GelC10G00226 | GT29 |
|  |  |  | GelC10G00227 | GT30 |
|  |  |  | GelC10G00228 | GT31 |
|  |  |  | GelC10G00230 | GT32 |
|  |  |  | GelC10G00232 | GT33 |
|  |  |  | GelC09G00742 | GT27 |
|  |  |  | GelC10G01043 | GT34 |
|  |  |  | GelC11G00725 | GT35 |
|  |  |  | GelC11G00730 | GT36 |
|  |  |  | GelC11G00732 | GT37 |
|  |  |  | GelC12G00737 | GT38 |
|  |  |  | GelC12G00738 | GT39 |
|  |  |  | GelC13G00037 | GT40 |
|  |  |  | GelC13G00141 | GT41 |
|  |  |  | GelC13G00835 | GT42 |
|  |  |  | GelC14G00261 | GT43 |
|  |  |  | GelC14G00345 | GT44 |
|  |  |  | GelC14G00858 | GT45 |
|  |  |  | GelC16G00686 | GT46 |

**Table S11.** Information of protein structure modeling.

| **Protein** | **Template** | **Seq Identity** |
| --- | --- | --- |
| GT16 | 6JEL | 46.75% |
| GT17 | 6JEL | 45.99% |
| GT18 | 8CHD | 50.69% |
| GT24 | 7VEJ | 40.13% |
| GT42 | 7VEJ | 44.72% |
| GT44 | 8CHD | 43.24% |
